# *N*-acyl Amides from *Neisseria meningitidis* and Their Role in Sphingosine Receptor Signaling

**DOI:** 10.1002/cbic.202200490

**Published:** 2022-10-13

**Authors:** Wooyoung Cho, Autumn G. York, Rurun Wang, Thomas P. Wyche, Grazia Piizzi, Richard A. Flavell, Jason M. Crawford

**Affiliations:** a Department of Chemistry, Yale University, New Haven, CT, USA; b Institute of Biomolecular Design & Discovery, Yale University, West Haven, CT, USA; c Department of Immunobiology, Yale University School of Medicine, New Haven, CT, USA; d Exploratory Science Center, Merck & Co., Inc., Cambridge, MA, USA; e Howard Hughes Medical Institute, Yale University School of Medicine, New Haven, CT, USA; f Department of Microbial Pathogenesis, Yale University School of Medicine, New Haven, CT, USA

**Keywords:** GPCR signaling, Host-microbe interaction, Natural products, N-acyl amides, Neisseria

## Abstract

*Neisseria meningitidis* is a Gram-negative opportunistic pathogen that is responsible for causing human diseases with high mortality, such as septicemia and meningitis. The molecular mechanisms *N. meningitidis* employ to manipulate the immune system, translocate the mucosal and blood-brain barriers, and exert virulence are largely unknown. Human-associated bacteria encode a variety of bioactive small molecules with growing evidence for *N*-acyl amides as being important signaling molecules. However, only a small fraction of these metabolites has been identified from the human microbiota thus far. Here, we heterologously expressed an *N*-acyltransferase encoded in the obligate human pathogen *N. meningitidis* and identified 30 *N*-acyl amides with representative members serving as agonists of the G-protein coupled receptor (GPCR) S1PR4. During this process, we also characterized two mammalian *N*-acyl amides derived from the bovine medium. Both groups of metabolites suppress anti-inflammatory interleukin-10 signaling in human macrophage cell types, but they also suppress the pro-inflammatory interleukin-17A+ population in T_H_17-differentiated CD4^+^ T cells.

## Graphical Abstract

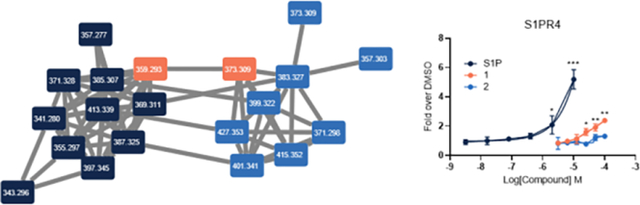

This study expands upon the biosynthetic and functional diversity of *N*-acyl amides through structural characterization of previously undescribed *N*-acyl amides from bacterial and mammalian origin and functional characterization of such molecules to signal through G-protein coupled receptor S1PR4, downregulate IL-10 in a macrophage model, and decrease IL17A+ population in T_H_17 cells.

## Introduction

The *Neisseria* genus is one of the most abundant groups in the human oral and nasopharyngeal microbial communities.^[1]^ While they mostly reside as commensals, particular species of *Neisseria*, such as *Neisseria meningitidis* and *Neisseria gonorrhoea*, have been characterized as opportunistic pathogens. *N. meningitidis* causes severe disease pathologies such as septicemia and meningitis when they enter the bloodstream by traversing the mucosal barrier and invade the cerebrospinal fluid (CSF) by translocating the blood-brain barrier.^[1]^ Interestingly, humans are the only known reservoir for *N. meningitidis*.^[2]^ In part due to a lack of animal studies for this reason, there is much left to be elucidated about this bacterium’s virulence mechanisms in humans. From genomic studies, it is observed that classical “virulence genes” are either absent in the *N. meningitidis* genome,^[2–3]^ or if present, are common to both *N. meningitidis* and purely commensal species of *Neisseria*.^[4–5]^ Pathogens, especially obligate human pathogens such as *N. meningitidis*, have been shown to exploit nutrients from the human host as a mechanism to promote virulence and survival,^[6]^ and it is reasonable to hypothesize that *N. meningitidis* has developed ways to metabolically adapt to the human host. In addition, *N. meningitidis* has been identified to increase sialic acid levels on its outer membrane, “mimicking” the membrane of eukaryotic cells as a method to evade lysis by the complement pathway.^[2, 7]^ Studying the metabolic products of *N*. *meningitidis* could provide further insights into its virulence mechanisms.

*N*-acyl amides are structurally and functionally diverse molecules that are ubiquitous in nature and biosynthesized by both eukaryotes and prokaryotes.^[8–10]^ They serve various signaling roles in human health and disease, including in inflammation, cell migration, diabetes, cancer, obesity, and pain sensation,^[11–13]^ and there is growing importance of *N*-acyl amides as human signaling molecules.^[14–16]^ One major way *N*-acyl amides mediate signal transduction is through G-protein coupled receptors (GPCRs), the largest group of membrane receptors that respond to a variety of molecular signals. Cohen *et al.* has shown that many of the bacterially-derived *N*-acyl amides that signal through GPCRs were structurally similar to the endogenous ligands of GPCRs, suggesting “molecular mimicry” by the bacteria.^[17]^ We sought to expand upon this hypothesis by analyzing a gene in *N. meningitidis* that is predicted to biosynthesize *N*-acyl amides. Here, we characterized a family of *N*-acyl amides encoded by this gene and assessed their immunological and GPCR signaling activities in human tissues.

## Results and Discussion

Brady and co-workers previously reported a family of human-derived microbial *N*-acyl synthase (*hm*-NAS) proteins, belonging to the PFAM13444 protein family, which was present in over 90% of the patient samples analyzed in their study.^[17]^ We searched for homologs of this family of *hm*-NASs in *N. meningitidis*. A single gene annotated as the PEP-CTERM/exosortase system-associated *N*-acyl amino acid synthase, which comprises a family of *N*-acyl amino acid synthases that is widely distributed among *Proteobacteria* and found to produce long-chain *N*-acyl amides,^[18]^ was identified in *N. meningitidis* using BLASTP analysis.

**Figure 1.**
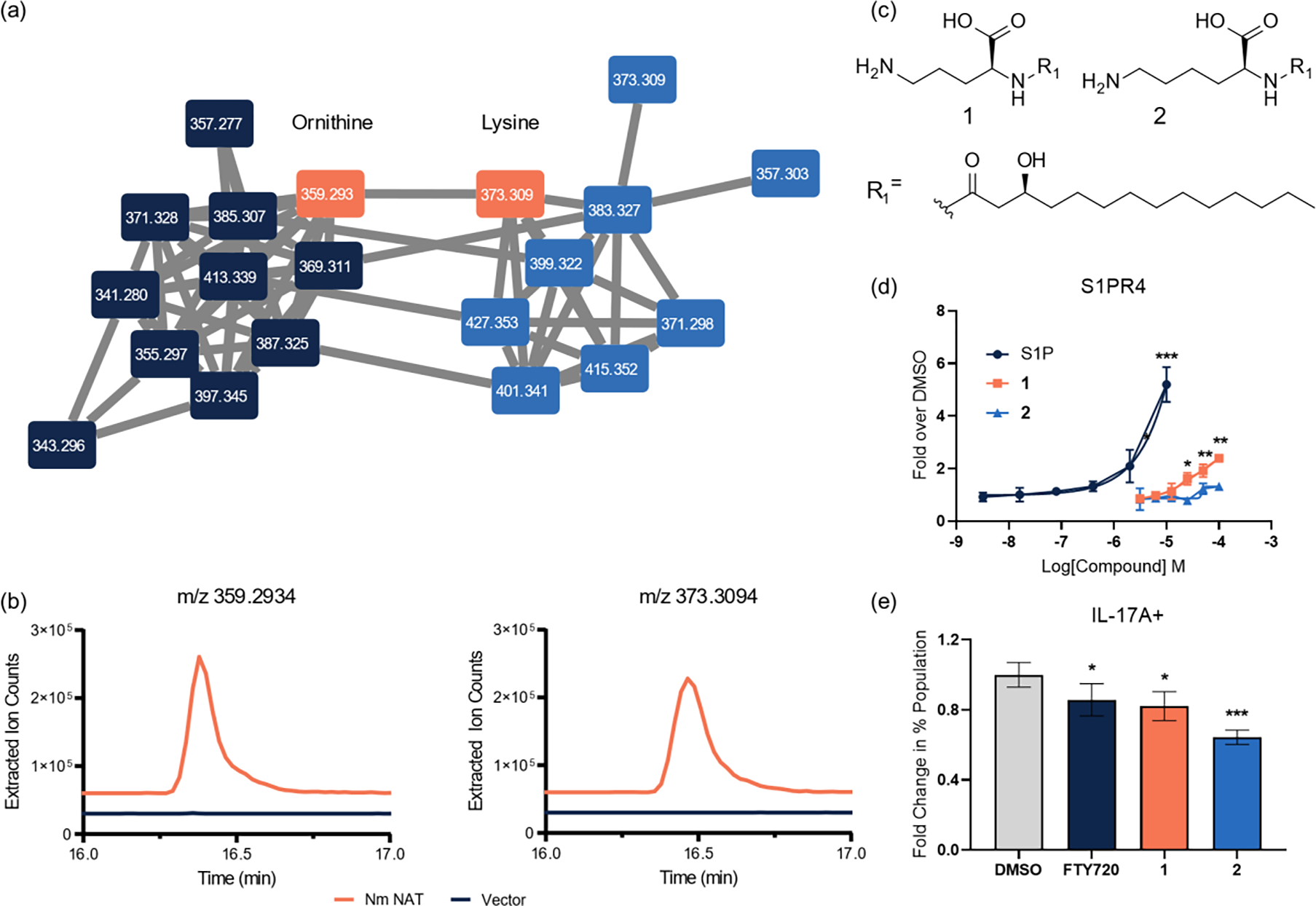
Identification of *N*-acyl amides **1** and **2** from *Nm*NAT and bioactivity testing. (a) Molecular networking of 20 of the 30 identified *N*-acyl ornithines (left, navy) and lysines (right, blue), where nodes are connected by tandem-MS patterns. (b) Production levels of **1** and **2** compared to the vector (pET28a) control. (c) New bacterially-derived metabolites **1** (*m/z* 359) and **2** (*m/z* 373). (d) Dose-dependent activation of S1PR4 by **1** and **2** observed in a PRESTO-Tango assay, quantified as fold over DMSO vehicle control (relative luminescence units (RLU) of compound divided by the DMSO RLU). Positive control ligand sphingosine-1-phosphate (S1P) is shown. (e) Change in percentages of IL-17A+ cells, quantified as fold over DMSO vehicle control in T_H_17 polarized cells from an IL-17A^Katushka^ IL-10^eGFP^ FoxP3^RFP^ reporter assay system in CD4^+^ T cells. Error bars represent SD. Two-tailed t-test. *p < 0.05, ** p < 0.01, *** p < 0.001, **** p < 0.0001.

The catalytic region annotated as the acyltransferase was heterologously expressed in *Escherichia coli* BAP1 to identify putative *N*-acyl amide products using liquid chromatography-quadrupole time-of-flight mass spectrometry (LC-QTOF-MS). Upon analysis of the high-resolution data, 30 peaks unique to the *N. meningitidis N*-acyltransferase (*Nm*NAT) were identified. Tandem MS analysis of these metabolites was supportive of their having *N*-acyl amide architectures, with diagnostic fragmentation at the amide bond. The peaks appeared as 15 pairs 14 Daltons apart with similar retention time patterns. The pairs were identified to be *N*-acyl-ornithines and *N*-acyl-lysines, which clustered as related molecular families by molecular networking (Figure 1a).^[19–20]^ The most abundant pair of metabolites, which were inconsistent with any previously described metabolites, were isolated and characterized by two-dimensional NMR spectroscopy. Analysis of the spectra revealed their planar structures as *N*-3-hydroxymyristoyl-ornithine **1** (*m/z* 359) and *N*-3-hydroxymyristoyl-lysine **2** (*m/z* 373) (Fig. 1b, Fig. 1c, Fig. S1, Fig. S2, Table S1).

To establish the absolute configurations of these metabolites, we conducted Marfey’s amino acid and Mosher ester analyses.^[21–23]^ Marfey’s analysis established that the ornithine and lysine head groups were in the *S*-configuration (L-amino acids), and Mosher ester analysis revealed that the 3-hydroxy groups on the acyl chains were similarly in the *S*-configuration for both **1** and **2** (Fig. S3, Fig. S4). This suggests that the fatty acid side chains are derived from β-oxidation, which typically proceeds through the *S*-β-hydroxy-configuration, rather than fatty acid synthesis, which normally proceeds through the *R*-β-hydroxy-configuration.^[24]^ These studies establish *N*-acyl-amides **1** and **2** as *N*-(*S*)-3-hydroxymyristoyl-L-ornithine and *N*-(*S*)-3-hydroxymyristoyl-L-lysine, which are previously uncharacterized bacterial metabolites. We synthesized metabolites **1** and **2** for structural and biological analysis, and co-injection studies with the natural materials confirmed their structures (Fig. S5). With confirmation of the major pair **1** and **2**, we could deduce the proposed structures of the other 28 related *Nm*NAT-derived ions by tandem MS, which includes the planar *N*-3-hydroxypalmitoyl-ornithine and *N*-3-hydroxypalmitoyl-lysine members previously identified by the Brady group.^[17]^ Acyl chains for the remaining ions varied from 14–18 carbons in length, with a single unit of unsaturation, single hydroxylation, or both (Table 1, Fig. S8, Fig. S9, Table S3).

*N*-acyl amides, specifically those derived from human commensal bacteria, have been shown to signal through a variety of GPCRs (G2A, GPR119, PTGER4).^[17]^ In particular, the *N*-3-hydroxypalmitoyl-ornithine and *N*-3-hydroxypalmitoyl-lysine were found to selectively activate sphingosine-1-phosphate receptor 4 (S1PR4).^[17]^ To determine whether the structurally similar compounds **1** and **2** could activate this GPCR in a β-arrestin-dependent manner, the compounds were tested for agonist activity for S1PR4 using the parallel receptorome expression and screening via transcriptional output, with transcriptional activation following arrestin translocation (PRESTO-Tango) assay compared to a positive control agonist, sphingosine-1-phosphate (S1P) (Fig. 1d).^[25]^ The results suggest the importance of the head group in the recognition of the ligand, as **1** exhibited significant agonist activity at 25 μM (EC_50_ 45 μM) while **2** activity was not statistically significant at up to 100 μM. These results are in agreement with the published result of *N*-3-hydroxypalmitoyl-ornithine displaying higher agonist activity over its lysine analog.^[17]^ Furthermore, the effective concentration of **1** required to activate the receptor was within the same order of magnitude as that of *N*-3-hydroxypalmitoyl-ornithine (EC_50_ 32 μM) despite the change in acyl chain length.

The GPCR target of these metabolites (S1PR4) is unique among the sphingosine receptor family in that its expression is largely exclusive to immune cells, and has been known to play a role in immune cell trafficking and differentiation.^[26–27]^ Activation of S1PR4 by the known ligand S1P has been reported to play a role in the downregulation of T cell proliferation and effector responses, such as decreased production of IFNγ, IL-2, and IL-4 and increased production of IL-10 in CD4^+^ T cells.^[28]^ To probe whether the activation of S1PR4 by compounds **1** and **2** had a similar effect in CD4^+^ T cells, we tested them using an IL-17A^Katushka^ IL-10^eGFP^ FoxP3^RFP^ triple reporter mouse model differentiated into T_H_17 cells with FTY720, a known S1PR agonist, as a positive control. Compounds **1** and **2** (at 50 μM), as well as FTY720 (at 5μM), led to significant decrease of the pro-inflammatory IL-17A+ population (Fig. 1e), corroborating the downregulation of immune activation in CD4^+^ T cells reported in the literature.^[28]^

**Figure 2.**
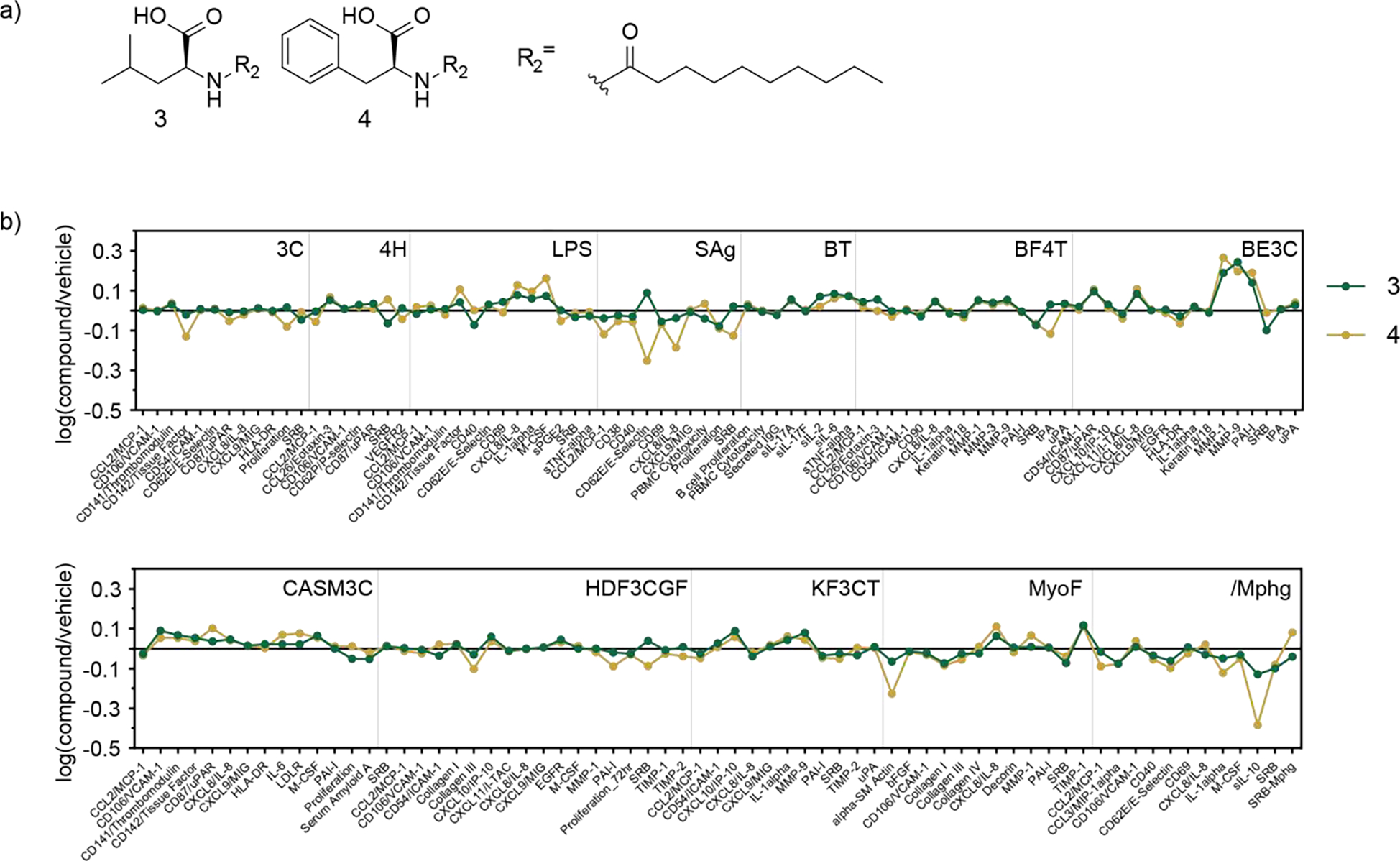
(a) Structures of **3** and **4**. (b) Eurofins DiscoverX BioMAP® diversity screen of compounds **3** and **4** in a panel of human primary cells (29 μM). Downregulation of sIL-10 in macrophages is shown in the /Mphg panel.

During the course of analyzing the *N. meningitidis* metabolites, we identified two additional *N*-acyl amides, compounds **3** and **4**, derived from the bovine brain-heart infusion (BHI) extract that exhibited moderate inhibitory activity against the Gram-positive bacterium *Bacillus subtilis* (Fig. 2a). Bioassay-guided fractionation of the BHI extract led to the active fraction containing **3** and **4** and ultraviolet-visible (UV) spectroscopy and tandem LC-MS analyses indicated the presence of an aromatic group and an amide linkage, respectively. Because these specific analogs had not been previously reported, we isolated and characterized their structures using NMR and Marfey’s analyses (Fig. S11, Fig. S12, Fig. S13, Fig. S14). These major molecules were similarly synthesized for functional analysis. Structurally related molecules had been identified in the brain tissues of rats^[9, 29]^ as well as from soil metagenomes.^[30]^ Bovine metabolites **3** and **4** displayed moderate activity against *B. subtilis* growth at IC_50_ values of 301 and 153 μM, respectively (Fig. S15). Although many bacterially-derived *N*-acyl amides have been identified to display antibacterial activity, the high concentrations required for this activity suggest it is not their primary function.^[10, 30–31]^ Structurally similar molecules have been found to be biosynthesized from mammalian sources, through enzymes such as mammalian fatty acid amide hydrolase (FAAH) detected in mouse liver and brain tissues, and the human *N*-acyl synthase PM20D1 that was found to regulate mitochondrial respiration in various human cells and modulate energy expenditure and glucose homeostasis in mice.^[32–33]^ We hypothesized that the mammalian compounds may have other important activities in human tissues, and consequently, we screened **3** and **4** at 29 μM for diverse immunological activities in the BioMAP® Phenotypic Profiling Assay system, a panel of 12 human primary cell-based co-culture systems, including venular endothelial cells, lung fibroblasts, and peripheral blood mononuclear cells (PBMCs), that model several tissues and diseases. Protein biomarker readouts provided an initial semi-quantitative triage for the effect of the two metabolites on the different cell systems. Treatment of **4** led to decreased levels of interleukin-8 (IL-8) and E-selectin in venular endothelial cells co-cultured with PBMCs. Treatment of **3** and **4** caused an increase in matrix metallopeptidase 1 (MMP-1), matrix metallopeptidase 9 (MMP-9), and plasminogen activator inhibitor-1 (PAI-1), all of which are associated with tissue remodeling. Additionally, treatment with **3** and **4** led to significantly decreased levels of secreted interleukin-10 (sIL-10) levels in the system of macrophages co-cultured with venular endothelial cells which models Th1 type inflammation (Fig. 2b), and thus we selected this activity for follow up validation.

**Figure 3.**
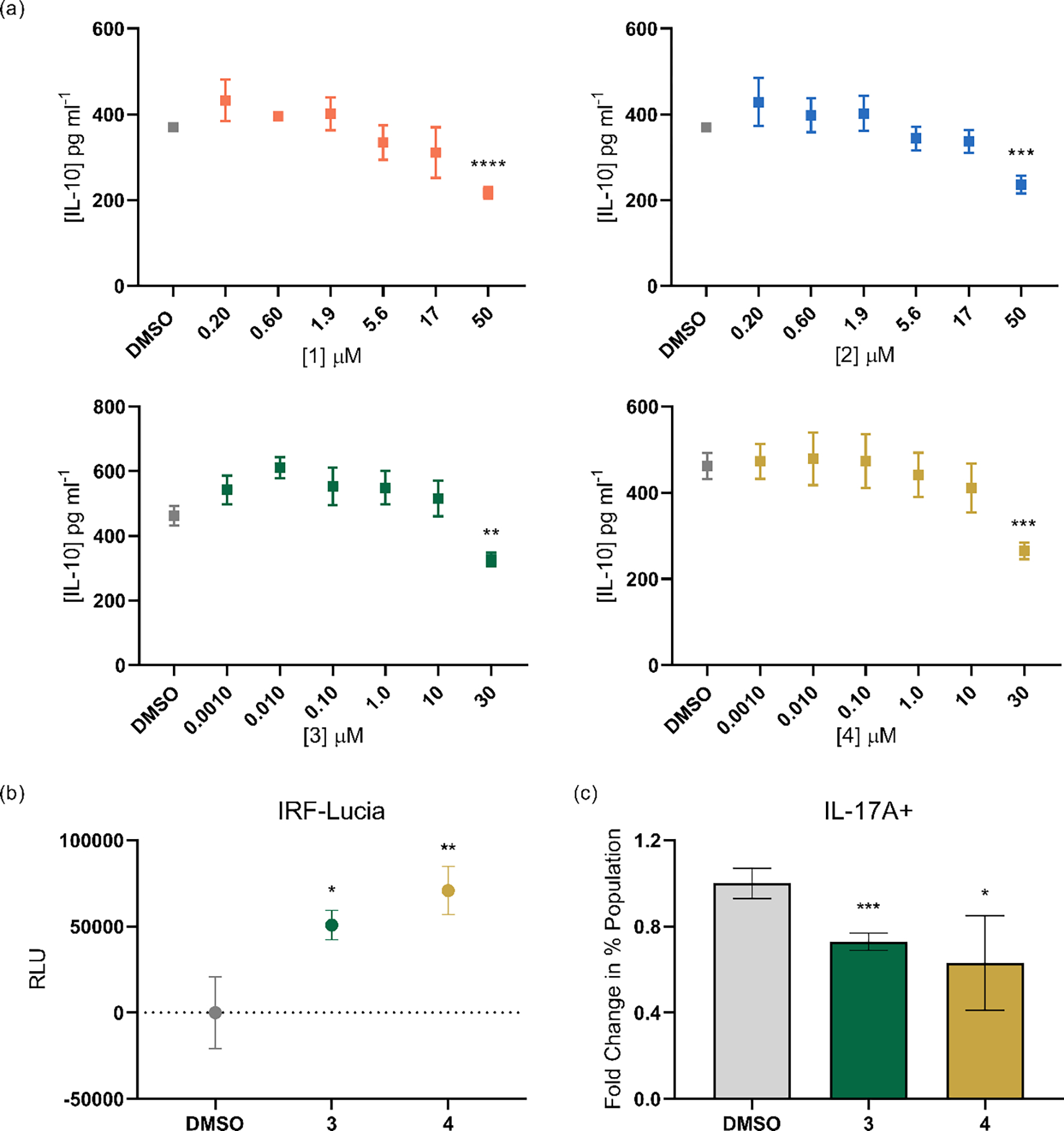
(a) Human IL-10 quantification of **1**-**4** by ELISA. (b) ISG levels, measured as secreted luciferase levels, from differentiated THP-1 ISG reporter cells (THP-1 Dual^™^) when treated with **3** and **4** (30 μM). Values represent RLU as luminescence units subtracted by the DMSO vehicle control value. (c) Change in percentages of IL-17A+ cells, quantified as fold over DMSO vehicle control in T_H_17 polarized cells, when treated with **3** and **4** (30 μM) in the CD4^+^ T cell IL-17A^Katushka^ IL-10^eGFP^ FoxP3^RFP^ reporter assay. Error bars represent SD. Two-tailed t-test. *p < 0.05, ** p < 0.01, *** p < 0.001, **** p < 0.0001.

To validate these findings, we screened **3**-**4** for IL-10 responses in human THP-1 cells differentiated into their macrophage-like state via ELISA. We also tested metabolites **1**-**2** in the macrophage model system, to test the potential cell-context dependence of the anti-inflammatory effect demonstrated in T cells. For metabolites **1** and **2**, we observed significantly lower levels of IL-10 at 50 μM, which is in contrast to the literature-reported findings in CD4^+^ T cells (Fig. 3a).^[28]^ We confirmed that the decreased IL-10 levels were not a result of cell toxicity, as assessed by the lactate dehydrogenase (LDH) release assay (Fig. S16), and we did not observe an effect on general indicators of inflammation, including NF-κB or interferon (IFN) responses, at concentrations up to 100 μM (THP-1-Dual^™^ Invivogen reporter system, data not shown). These studies suggest that bacterial metabolites **1**-**2** affect IL-10 regulation by a different pathway or that S1PR4 agonists have immune cell context-dependent activities. Mammalian compounds **3** and **4** also induced downregulation of anti-inflammatory IL-10 levels at 30 μM, which was consistent with the BioMAP® results, although these metabolites significantly increased pro-inflammatory interferon stimulated gene (ISG) responses (30 μM, THP-1 Dual^™^) (Fig. 3a, 3b). We also tested compounds **3** and **4** in the IL-17A^Katushka^ IL-10^eGFP^ FoxP3^RFP^ mouse model system for potential cell context-dependent differential activity, as observed with compounds **1** and **2** in CD4^+^ T cells and THP-1 cells. Similarly, treatment of compounds **3** and **4** in T_H_17 differentiated cells resulted in a significantly decreased IL-17A+ population (Fig. 3c), suggesting that these compounds promote polarization to an anti-inflammatory response in CD4^+^ T cells but pro-inflammatory response in the macrophage model system.

Our studies here expand the structural and functional repertoire of both bacterial and mammalian *N*-acyl amides. *N. meningitidis N*-acyltransferase is capable of producing 30 *N*-acyl-amide analogs, the majority of which have not previously been characterized, belonging to two structural groups. Detailed structural characterization of the major members of these two groups (**1**-**2**) defines their absolute configurations and suggests that their fatty acid appendages are derived from the fatty acid β-oxidation pathway. These bacterial molecules are agonists of the human S1PR4 GPCR signal transduction pathway and promote an anti-inflammatory response through the decreased levels of IL17A+ population in T_H_17 cells. However, they unexpectedly down-regulate the anti-inflammatory cytokine IL-10 in a human macrophage model system, suggesting that the pro- and anti-inflammatory responses can be dependent on the cellular context.

Intriguingly, mammalian *N*-acyl amides are convergently produced through the peptidase M20 domain containing 1 (PM20D1) enzyme, which catalyzes the condensation of free fatty acids and amino acids, with highest selectivity for phenylalanine and leucine, respectively.^[32]^ Consistent with this reported selectivity, we identified two new mammalian *N*-acyl amide members (**3**-**4**) with differing acyl chain lengths derived from bovine extracts. These metabolites down-regulated the anti-inflammatory cytokine IL-10 and stimulated the IFN signaling pathway in the macrophage-model system, but they decreased the pro-inflammatory IL-17A+ population in T_H_17 cells, highlighting differences in cytokine responses in different immune cell types. In sum, our studies suggest that the structural diversity of *N*-acyl-amides is greater than appreciated in bacteria and their hosts and add to the growing body of evidence that *N*-acyl amides are key small molecule mediators of immune system function.

## Experimental

### Instrumentation

Low-resolution liquid chromatography-mass spectrometry (LC-MS) data were collected using an Agilent 6120 single quadrupole LC-MS system and a Phenomenex Kinetex 5 μm C18 (250 × 4.6 mm) column with a gradient of water:acetonitrile 10–100% acetonitrile (MeCN) containing 0.1% formic acid, 0–30 min at 0.7 ml min^−1^ flow rate.

High-resolution electrospray ionization mass spectrometry (HR-ESI-MS) data were collected using an Agilent 6546 LC/quadrupole time-of-flight (Q-TOF) mass spectrometer coupled to an Agilent 1290 Infinity II high performance liquid chromatography (HPLC) system. The Kinetex 5μ C18 100 Å column (250 × 4.6 mm) was used with a water:acetonitrile mobile phase containing 0.1% formic acid and a gradient of 5 to 100% acetonitrile from 0 to 30 min at 0.7 ml/min.

Isolation of metabolites was performed at indicated gradients on an Agilent Prepstar HPLC instrument with a preparative Agilent Polaris C18-A 5μ (250 × 21.2 mm) column, semi-preparative Phenomenex Luna C18(2) 100 Å (250 × 10 mm) column, and semi-preparative Agilent phenyl-hexyl 5 μ (250 × 9.5 mm) column, using a water:acetonitrile solvent system containing 0.01% trifluoroacetic acid (TFA) as the mobile phase.

Nuclear magnetic resonance (NMR) spectra (^1^H, gCOSY, gHSQC, gHMBC, gTOCSY) were collected on an Agilent 600 MHz NMR spectrometer with a cold probe in a 3-mm sample tube, or a Bruker 400 MHz NMR spectrometer with a broadband probe in a 5-mm sample tube. The chemical shifts for the spectra were recorded as δ values (ppm) referenced to the respective solvent signals.

### Heterologous expression of *N. meningitidis N*-acetyltransferase gene in *Escherichia coli*

#### *N*-acetyltransferase: GenBank Accession SPY04274.1; Region 191–450

The *N*-acetyltransferase gene was codon optimized and synthesized by GeneWIZ. The gene was appended with BamH1 and Xho1 at the N- and C- termini, respectively, and ligated into pET28a at the corresponding restriction sites. The plasmid was transformed into *Escherichia coli* Mach1, and positive colonies were screened by plating on Luria-Bertani agar plates supplemented with kanamycin (50 μg ml^−1^). After sequence validation, the plasmids were then transformed into *E. coli* Bap1 for expression. Three biological replicates of *E. coli* Bap1:*Nm*NAT clones were inoculated into 5 ml LB broth containing kanamycin (50 μg ml^−1^) and grown aerobically overnight at 37⁰C and 250 rpm. 100 μl of the overnight cultures was used to inoculate three corresponding cultures in LB with kanamycin. When the cultures reached optical density (OD_600_) of 0.6, they were supplemented with isopropyl β-D-1-thiogalactopyranoside (IPTG) (100 μM), and grown for an additional 48 h at 30⁰C and 225 rpm. Each culture was extracted with 6 ml ethyl acetate, and 4 ml of the organic extract was taken to be dried *in vacuo*. The dried extracts were resuspended in 100 μl of methanol and analyzed with single-quadrupole LC-MS. In parallel, triplicate cultures of *E. coli* Bap1 transformed with an empty pET28a were treated with the same methods for comparison of LC-MS traces.

### Isolation of metabolites

#### Compounds **1** and **2**

Sequence validated *E. coli* Bap1:*Nm*NAT clones were inoculated into 4 5-ml LB broth containing 50 μg ml^−1^ kanamycin and grown at 37⁰C and 250 rpm overnight. Each 5 ml culture was used to inoculate 4 1-L LB cultures and grown until the cultures reached OD_600_ of 0.6. The cultures were then supplemented with IPTG (100 μM) and induced for 48 h at 30⁰C and 225 rpm. Each liter of culture was extracted twice with equal volumes of ethyl acetate, and the organic layer was dried under rotary evaporation. The crude extract was dissolved in a solvent mixture of 20% acetonitrile, 10% methanol, 70% water containing 0.01% TFA. Compounds **1** and **2** eluted together at T_R_=37 min in the 20% to 80% aqueous acetonitrile gradient over 0 to 60 minutes using the semi-preparative Phenomenex Luna C18(2) 100 Å (250 × 10 mm) column. 3 mg of the semi-pure mixture was collected and analyzed by NMR.

#### Compounds **3** and **4**

6 L of Brain Heart Infusion (BHI) was extracted into equal volumes of ethyl acetate twice, and dried by rotary evaporation. The crude extract was dissolved in methanol, mixed with 3 g of Celite®110, and dried by rotary evaporation. The resulting extract-Celite®110 mixture was loaded on the Waters Sep-Pak® Vac 35cc (10g) C_18_ cartridge and separated into 5 fractions using a step gradient with 20%, 40%, 60%, 80%, and 100% aqueous acetonitrile. Fraction 3 (60% aqueous acetonitrile), which showed antibacterial activity against *Bacillus subtilis*, was further separated into 15 fractions using a reversed-phase HPLC system with the preparative C18 column and a gradient of 10–100% aqueous acetonitrile containing 0.01% TFA from 0 to 30 minutes (flow rate: 8 ml min^−1^). Fraction 9 contained two major peaks corresponding to the masses of compounds **3** and **4**. The compounds were purified using the semi-preparative Agilent phenyl-hexyl 5 μ (250 × 9.5 mm) column in a 30-min gradient of 50–100% aqueous MeCN containing 0.01% TFA (Flow rate: 4 ml min^−1^). Compound **3** T_R_= 9.1 min; Compound **4** T_R_=9.9 min.

### Structural determination of *N*-acyl amide families

The triplicate cultures of *E. coli* Bap1:*Nm*NAT were analyzed by LC-MS, Q-TOF, and Tandem MS. Predicted structures **3** and **4** were synthesized through liquid-phase peptide synthesis, using *N*_δ_-Boc-*N*_α_-Fmoc-L-ornithine-OH (Alfa Aesar) or *N*_α_-Fmoc-*N*_ε_-Boc-L-lysine-OH (ACROS Organics) and ±3-hydroxymyristic acid (TCI). The following adjustments to the protocol were made: 2 eq. benzotriazole-1-yloxytris(dimethylamino)phosphonium hexafluorophosphate (BOP), 2 eq. hydroxybenzotriazole (HOBt) hydrate, 4 eq. *N*,*N*-Diisopropylethylamine (DIPEA) in 2 ml dimethylformamide (DMF) was added to 2 eq. of ± 3-hydroxymyristic acid, which was then added to the deprotected *N*_δ_-Boc-L-ornithine-OH or *N*_ε_-Boc-L-lysine-OH, and equal volumes of dichloromethane (DCM) and TFA containing 5% triisopropyl silane (TIPS) was used as the cleavage mixture.

Compounds **3** and **4** were synthesized through a solid-phase peptide synthesis method according to Coin *et al.*^[35]^ using Fmoc-L-phenylalanine-Wang resin or Fmoc-L-leucine-Wang resin and decanoic acid. The following adjustments were made: 2 eq. of BOP and 4 eq. DIPEA were added to the decanoic acid, and this mixture was added to the L-phenylalanine-Wang resin or L-leucine-Wang resin, and 95% TFA, 5% TIPS cleavage mixture was used.

Crude Bap1:*Nm*NAT ethyl acetate extract and synthetic **1** and **2** were analyzed separately on Q-TOF, and 1:1 mixtures of the crude natural and synthetic compounds were analyzed, which exhibited a co-elution pattern.

Crude BHI ethyl acetate extract and synthetic **3** and **4** were analyzed separately on single-quadrupole LC-MS, and 1:1 mixtures of the crude natural and synthetic compounds were analyzed, which also exhibited a co-elution pattern.

### Marfey’s analysis

Isolated metabolites **1**-**4** were hydrolyzed in 500 μl of 6N hydrochloric acid (HCl) at 110⁰C for 1 h, and the reaction was dried under nitrogen gas. The hydrolysate was resuspended in 3 ml of distilled water and dried *in vacuo* to remove excess acid. Standard D- and L-amino acids and hydrolysates were treated with 50 μl of *N*_α_-(2,4-dinitro-5-fluorophenyl)-L-alaninamide (10 mg ml^−1^ in acetone) and 100 μl of 1N sodium bicarbonate (NaHCO_3_). The mixture was heated at 80⁰C for 3 min, cooled to room temperature, quenched with 50 μl HCl (2N), and reduced *in vacuo*. The derivatized amino acids were then dissolved in 100 μl of H_2_O/MeCN (50/50) for analysis on the single-quadrupole LC-MS system.

### Mosher ester analysis

A sample containing a mixture of natural compounds **1** and **2** was purged with nitrogen gas overnight and dissolved in 250 μl of dried pyridine-*d*_5_. Dimethylaminopyridine (DMAP) (0.5 mg) and 5 μl of *S*- or *R*-α-methoxy-(trifluoromethyl)phenylacetyl chloride (MTPA-Cl) solution (2% v/v) were added and incubated for 18 h. The reaction mixtures were dried *in vacuo* and resuspended in CDCl_3_. ^1^H NMR spectra was collected on an Agilent 600 MHz NMR in a 3-mm tube, and the Δδ_S-R_ values were quantified to determine the absolute configuration.

### GPCR PRESTO-Tango Assay

GPCR screening was performed according to the protocol reported by Chen *et al.*^[36]^ Briefly, HTLA cells (a HEK293-derived cell line containing a stably integrated tTa-dependent luciferase reporter and a β-arrestin2-TEV fusion gene) maintained in DMEM containing 10% fetal bovine serum (FBS) and 1% Penicillin/Streptomycin were seeded in 96-well tissue culture plates (Eppendorf) and transfected with 200 ng of S1PR4 plasmid (Addgene plasmid #66499)^[25]^ for 20 h. The medium was then replaced with Dulbecco’s Modified Eagle Medium (DMEM) with 20 mM *N*-2-hydroxyethylpiperazine-*N*-ethanesulfonic acid (HEPES) buffer and 1% Penicillin/Streptomycin, and independently treated with compounds **1** and **2**, sphingosine-1-phosphate (S1P) (Fisher Scientific), and dimethyl sulfoxide (DMSO) solvent vehicle after 2 h. After 20 h of treatment, the luminescence was read by incubating each well for 20 min with 50 μl/well of Bright-Glo solution (Promega) diluted 20-fold in Dulbecco’s phosphate-buffered saline (DPBS) with 20 mM-HEPES. The luminescence was measured using Perkin Elmer EnVision 2100 plate reader.

### BioMAP® Diversity ES Panel

Bovine metabolites **3** and **4** were analyzed using the Eurofins DiscoverX BioMAP® panel. The cell systems as well as stimuli used are: 3C system [HUVEC + (IL-1β, TNFα and IFNγ)], 4H system [HUVEC + (IL-4 and histamine)], LPS system [PBMC and HUVEC + LPS (TLR4 ligand)], SAg system [PBMC and HUVEC + TCR ligands (1x)], BT system [CD19+ B cells and PBMC + (α-IgM and TCR ligands (0.001x))], BF4T system [bronchial epithelial cells and HDFn + (TNFα and IL-4)], BE3C system [bronchial epithelial cells + (IL-1β, TNFα and IFNγ)], CASM3C system [coronary artery smooth muscle cells + (IL-1β, TNFα and IFNγ)], HDF3CGF system [HDFn + (IL-1β, TNFα, IFNγ, EGF, bFGF and PDGF-BB)], KF3CT system [keratinocytes and HDFn + (IL-1β, TNFα and IFNγ)], MyoF system [differentiated lung myofibroblasts + (TNFα and TGFβ)] and /Mphg system [HUVEC and M1 macrophages + Zymosan (TLR2 ligand)].

Abbreviations that are used are: Human umbilical vein endothelial cells (HUVEC), peripheral blood mononuclear cells (PBMC), human neonatal dermal fibroblasts (HDFn), B cell receptor (BCR), T cell receptor (TCR), and toll-like receptor (TLR). Compounds were dissolved in DMSO to a final concentration of ≤1% and added to the cells 1 h prior to addition of stimuli, and incubated for 24 h (with the exception of MyoF system: 48 h, BT system (soluble readouts): 72 h, BT system (secreted IgG): 168 h). The assays include appropriate drug controls, non-stimulated controls, and vehicle controls (0.1% DMSO) for each system.

### IL-10 ELISA, ISG, and NF-κB assays

THP-1-Dual^™^ (Invivogen) cells cultured in RPMI medium (Gibco 11875093) with 10% FBS (Gibco) and 1% Penicillin/Streptomycin (Gibco) were seeded into 96-well plates and differentiated into macrophage-like state by incubation with 50 nM phorbol 12-myristate 13-acetate (PMA) (Promega, V1171–5mg) for 72 h. The cells were then treated with compound **1** or **2** (50 μM, 17 μM, 5.6 μM, 1.9 μM, 0.60 μM, 0.020 μM) or compound **3** or **4** (30 μM, 10 μM, 1 μM, 0.1 μM, 0.01 μM, 0.001 μM) in DMSO (final concentration 0.1%) or 0.1% DMSO 1 h prior to addition of 100 ng ml^−1^ LPS (Invivogen) in triplicate wells. After incubation for 24 h, the culture supernatants were collected for the IL-10 enzyme-linked immunoassay (ELISA) (R&D systems, DY217B), which was conducted according to the manufacturer’s instructions.

THP-1-Dual^™^ cells contain two inducible promoters, an ISG54 minimal promoter that is fused to 5 IFN-stimulated response elements (ISREs), and an IFN-β minimal promoter that is fused to 5 replicates of the NF-κB transcriptional response element and 3 replicates of the c-Rel binding site. Upon stimulation of the promoter, the cells express a secreted luciferase reporter gene or the secreted embryonic alkaline phosphatase (SEAP) reporter gene for interferon stimulated genes (ISGs) or NF-κB readout, respectively. The supernatants collected from the IL-10 assays were incubated with the luciferase detection agent, QUANTI-Luc^™^ to observe ISG responses, or with the SEAP detection agent, QUANTI-Blue^™^ to detect NF-κB responses, according to the manufacturer’s instructions.

### T_H_17 CD4^+^ T cell experiments

Triple reporter mice (Foxp3^RFP^, IL-10^eGFP^, IL-17A^Katushka^) generated by the Flavell group were used in these studies.^[37]^ Naïve CD4^+^ T cells were isolated from the spleen and lymph nodes of 2 mice with the mouse naïve CD4^+^ T cell isolation Kit (Stemcell). Cells were labeled with CellTrace^™^ Violet (CTV) to monitor cell proliferation. The cells were cultured in Click’s medium supplemented with 5% FBS, 0.1 mM β-mercaptoethanol, Glutamax^™^ (Gibco), 100 U/mL Penicillin/Streptomycin, and differentiated using 10 μg/mL anti-CD3, 2 μg/mL anti-CD28, 10 μg/mL anti-IFNγ, and 10 μg/mL anti-IL-4, in addition to 20 ng/mL murine IL-23, 20 ng/mL murine IL-6, 0.5 ng/mL murine TGF-β1 for T_H_17. A DMSO vehicle or compound was added to the cultures after 1–3 days and cultured for an additional 3 days, followed by flow cytometry analysis.

### Cytotoxicity LDH Assay

THP-1 Dual^™^ cells were treated in the same conditions as the IL-10 assay, and the cytotoxicity was measured using CyQUANT^™^ LDH Cytotoxicity Assay Kit (Thermo Fisher Scientific). The assay was performed according to the manufacturer’s instructions, in which the supernatant of triplicate wells containing ~1×10^5^ cells and 0.1% DMSO was used to determine the spontaneous lactate dehydrogenase (LDH) release, and the cells grown in the same conditions were lysed to determine the maximum LDH release. Absorbance measurements at 490 nm and 680 nm were taken on a Perkin Elmer EnVision 2100 plate reader.

### Antimicrobial Assays

Compounds **1**-**4** were dissolved in DMSO to a concentration of 50 mM. Each compound was diluted in corresponding bacterial/yeast medium to yield final concentrations of 500, 250, 125, 62.5, 31.25, 15.625, 0 μM (1% final DMSO concentration) in triplicate wells of a 96-well microplate.

*B. subtilis*, *E. coli* DH5α, and *Pseudomonas aeruginosa* PA01 were grown in LB broth, *Staphylococcus aureus* was grown in BHI, and *Saccharomyces cerevisiae* was grown in yeast extract-peptone-dextrose (YPD) overnight. The bacterial/yeast cultures were diluted to OD_600_ of 0.5, then diluted further to yield 1e6 cells ml^−1^. 50 μl of the diluted bacterial cultures was added to each well containing compound or control.

The mixture was incubated overnight in a stationary 37⁰C incubator, with the exception of *B. subtilis* and *S. cerevisiae*, which were incubated in a stationary 30⁰C incubator. The OD_600_ for each antimicrobial assay was measured on a Perkin Elmer EnVision 2100 plate reader.

## Supplementary Material

supinfo

## Figures and Tables

**Table 1.** *N*-acyl amides detected from *Nm*NAT expression.

Acyl Chain	Orn	Lys

OH-C14:0	359.2934	373.3094
OH-C15:0	373.3086	387.3234
OH-C16:0	387.3246	401.3393
OH-C17:0	401.3381	415.3536
C14:0	343.2983	357.3132
C16:0	371.3279	385.3423
OH-C14:1	357.2746	371.2915
OH-C16:1	385.3071	399.3224
OH-C17:1	399.3218	413.3376
OH-C18:1	413.3387	427.3548
C14:1	341.2811	355.2962
C15:1	355.2959	369.3115
C16:1	369.3113	383.3275
C17:1	383.3278	397.3427
C18:1	397.3426	411.3589
