# Supplementary material for: N-acyl Amides from Neisseria meningitidis and Their Role in Sphingosine Receptor Signaling
Source: Chembiochem. Author manuscript; Available in PMC 2022 Dec 19. (PMC9762135; doi:10.1002/cbic.202200490)
Supplement: supinfo [file NIHMS1852871-supplement-supinfo.pdf]

GenBank Accession: SPY04274.1; Region 191-450

*Nm*NAT nucleotide sequence

ATGTCCCCCAGCCGTTTCAATCAAACGGGCCCCAAAATCGGCCTGAGCGTACGCCTTGCAGAAACC
CAAGCTGAAATCGAAGCCGCCCAAAGATTGCGCTATCAGGTTTTTGCCCAAGAGTTGGGGGCGGAA
ATCGAAAGCGATGACGGCCGTGATGTCGATCCTTATGATGAGCATTGCCACCACCTGCTTGCCTTCG
ACGATGCAACCGGCGAAGTTATCGGCTGCTACCGCCTGATTACCGAAGAAACCGCGAAAAAAGTCG
GCGGCTGGTACAGCGAGCATGAATTCGACCATGAGCCTTTGAAAGACATTCTGCCGCAAACCGTCG
AACTCGGTCGCGCCTGTACCCACCCGGACTACCGCAACGGCGGCTTGGTCATGCTGTTGTGGACCG
GTTTGGTCAAATTCATGAAAGACGAAAACCTGCGCTTTATGATTGGTTGCGGCAGTATCGAAATGCG
CGACGGCGGCAACGATGCGGCGGGCCTGTATCATGCTTTGAAAGACAAATACCTCGCTCCGGAACA
ATGGCGCGTCAAACCGCTCAACCCGCTCAAATGGGACAGCATCACGCCGTCTGAAAATCCGCCTGT
ACCCGCACTGATCAAAGGCTATCTCAAAGCAGGCGCATGGTTCTGCGGCGAGCCTTGCGTCGATGA
AGCATTCAACTGCGCGGATGTGCTGATCATGATGGACATCAGCCACCTTTCCGACCGCTACTTGCAG
CGTTTTGCCCCTAAAACCGATTCATAA

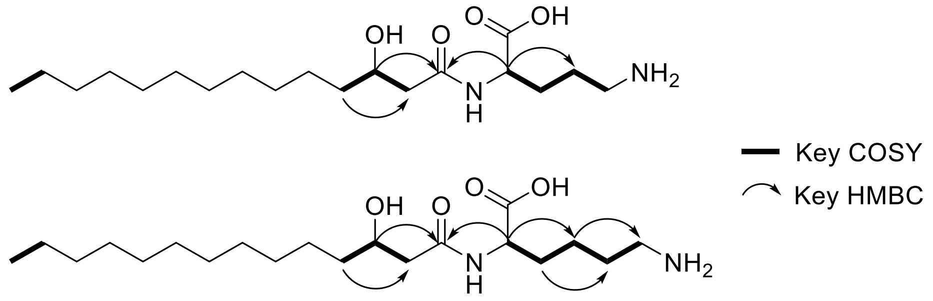

Figure S1. Key COSY and HMBC NMR correlations for **1** and **2**.

***N*-3-hydroxymyristoyl-L-ornithine (MH+ 359)** (MeOD, 600 MHz)

	NH	αH	βH	γH	δH	
N-3-hydroxymyristoyl		2.35	3.95	1.48		CH2 1.23-1.37<br>Terminal CH3 0.88
Orn		4.45	1.74<br>1.99	1.49	2.94	

	CO	αC	βC	γC	δC	
N-3-hydroxymyristoyl	172.92	43.16	68.38	36.87		Terminal<br>CH3 12.93
Orn	173.14	51.20	28.37	22.29	38.76	

***N*-3-hydroxymyristoyl-L-lysine (MH+ 373)** (MeOD, 600 MHz)

	NH	αH	βH	γH	δH	
N-3-hydroxymyristoyl		2.35	3.95	1.48		CH2 1.23-1.37

						Terminal CH3 0.88
Lys		4.42	1.92<br>1.70	1.49	1.66	εH 2.90

	CO	αC	βC	γC	δC	
N-3-hydroxymyristoyl	172.92	43.16	68.38	36.87		Terminal CH3 12.93
Lys	173.66	51.47	30.76	22.29	26.52	εC 39.09

Table S1. Chemical shifts of natural (bacterially-produced) **1** and **2** as a mixture identified by 1D- and 2D-NMR (gCOSY, gHSQC, gHMBC)

A

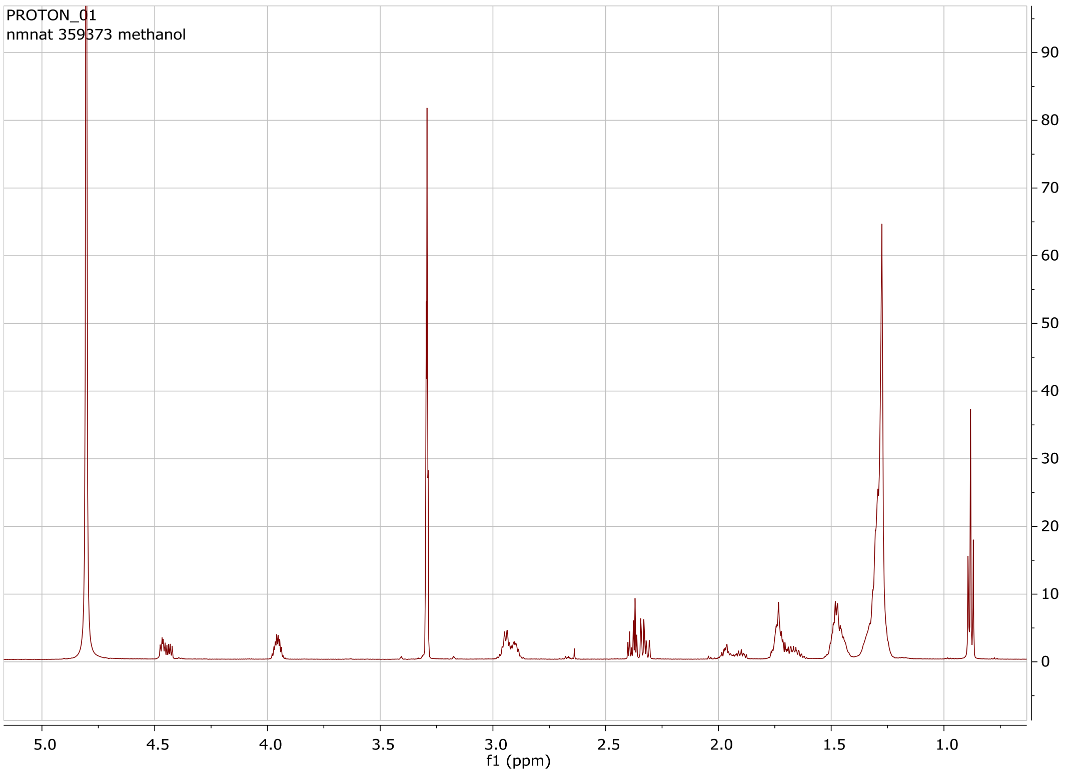

B

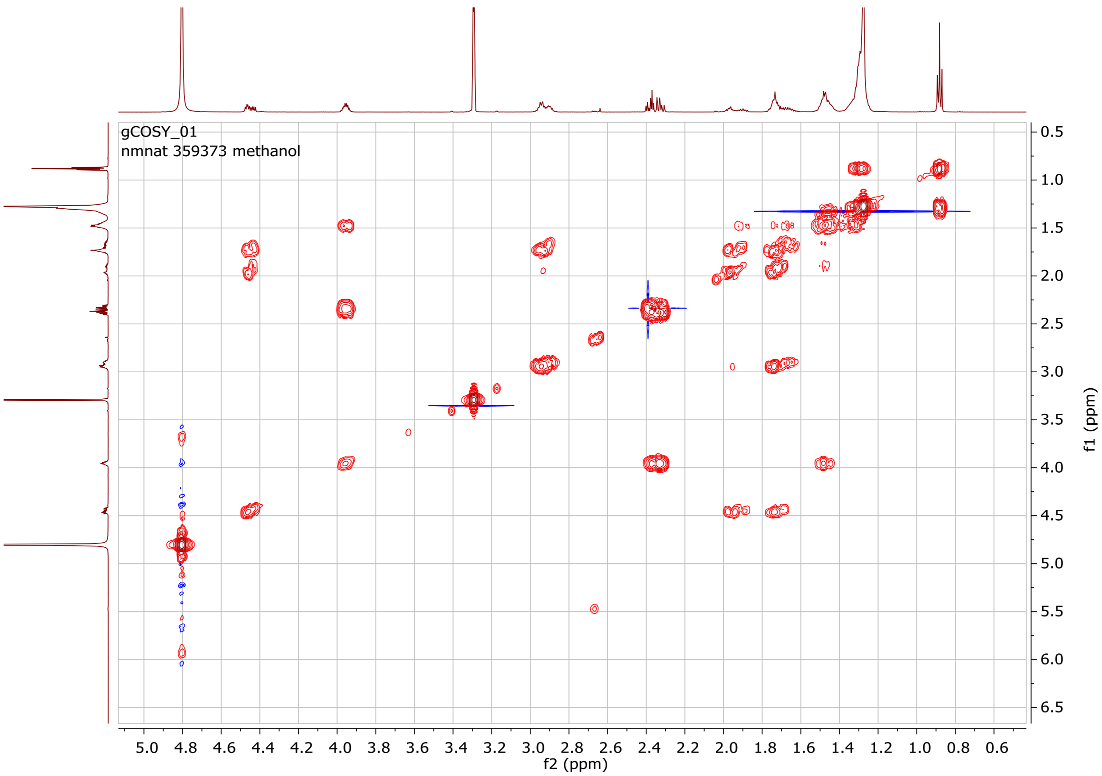

gCOSY_01
nmnat 359373 methanol
5.0 4.8 4.6 4.4 4.2 4.0 3.8 3.6 3.4 3.2 3.0 2.8 2.6 2.4 2.2 2.0 1.8 1.6 1.4 1.2 1.0 0.8 0.6
f2 (ppm)
0.5 1.0 1.5 2.0 2.5 3.0 3.5 4.0 4.5 5.0 5.5 6.0 6.5
f1 (ppm)

C

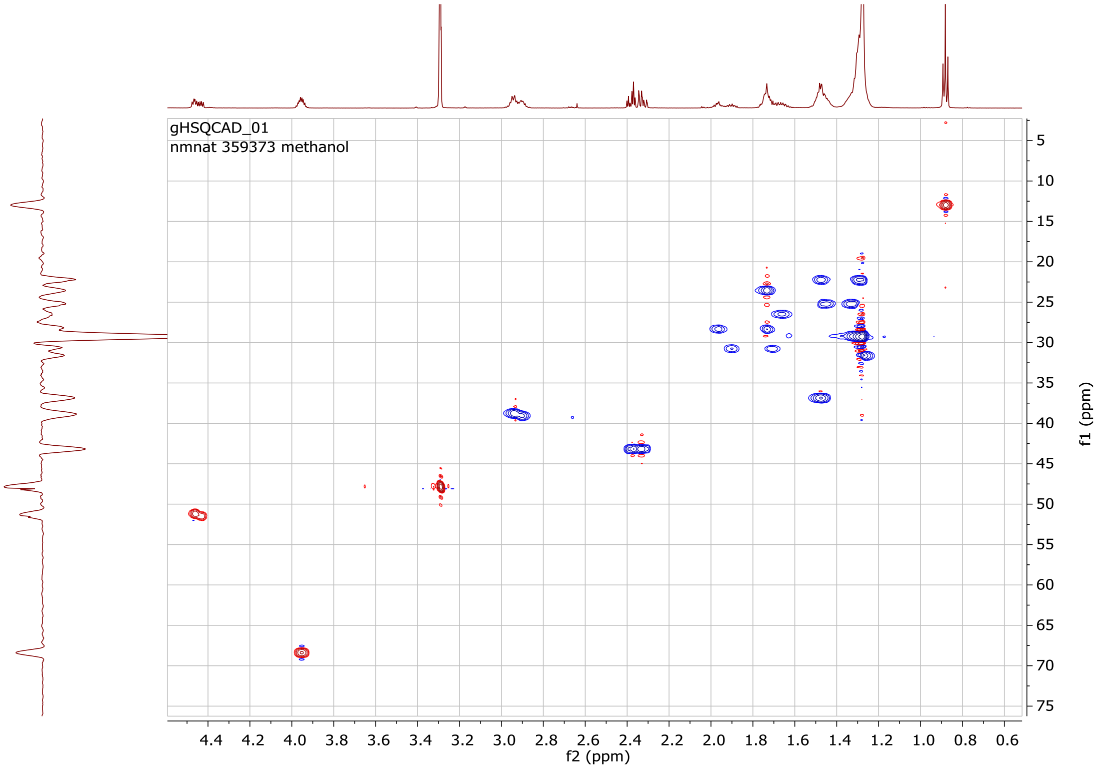

D

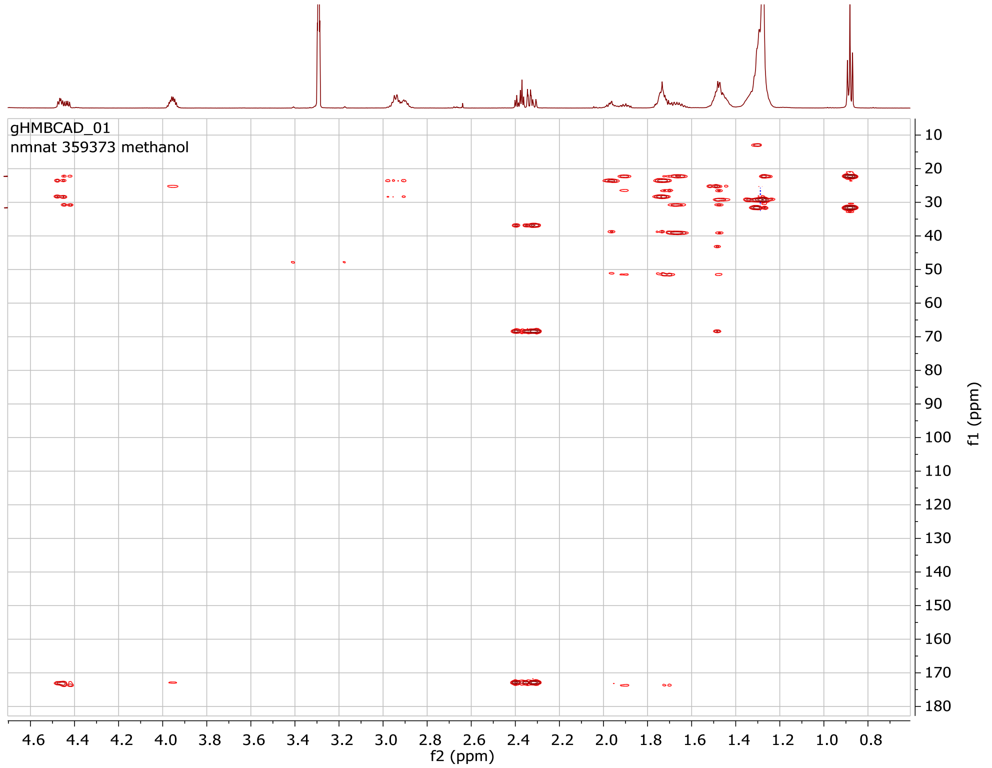

Figure S2. NMR spectra of natural **1** and **2** as a mixture (MeOD, 600 MHz)

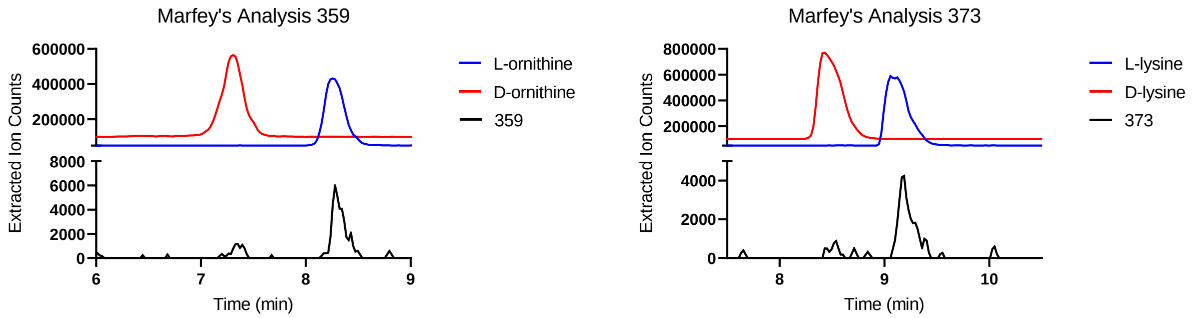

Figure S3. Marfey's analysis of **1** and **2**. Extracted ion chromatograms (*m/z* 385.1466, *m/z* 399.1623) of the Marfey's product of L-ornithine, D-ornithine, L-lysine, D-lysine, hydrolyzed **1**, hydrolyzed **2** are shown, both (**1** and **2**) of which align in retention time with that of the *S*- configuration of their respective amino acid head groups.

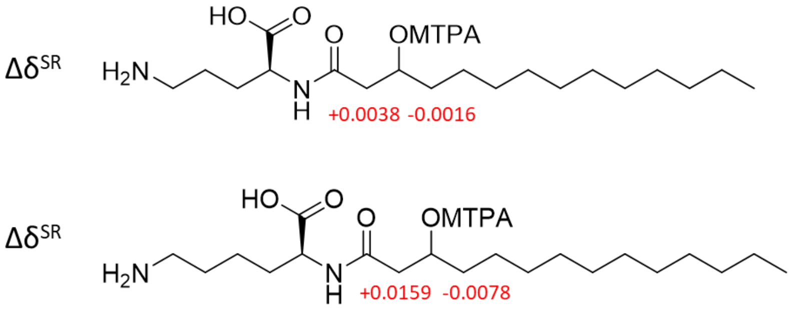

Figure S4. The $\Delta\delta_{S\text{-}R}$ in ppm for the MTPA esters of compounds **1** and **2**.

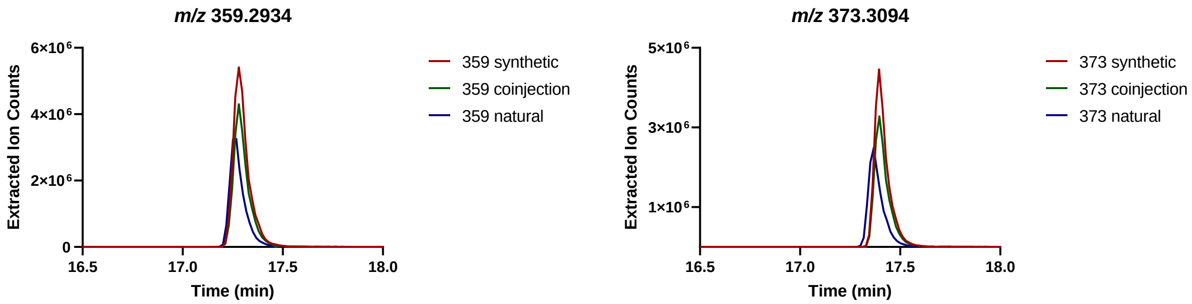

Figure S5. Extracted ion chromatogram of crude *Nm*NAT extract, synthetic, and co-injection of crude and synthetic **1** and **2**.

***N*-3-hydroxymyristoyl-L-ornithine (MH+ 359)** (DMSO, 600 MHz)

	NH	αH	βH	γH	δH	
N-3-hydroxymyristoyl		2.16	3.72	1.30, 1.26		CH2 1.20-1.32 Terminal CH3 0.82
Orn	7.84	4.02	1.58, 1.72	1.55	2.74	

	CO	αC	βC	γC	δC	
N-3-hydroxymyristoyl	171.13	44.10	67.95	37.34		Terminal CH3 14.40
Orn	173.92	52.51	29.27	24.26	38.99	

***N*-3-hydroxymyristoyl-L-lysine (MH+ 373)** (DMSO, 600 MHz)

	NH	αH	βH	γH	δH	
N-3-hydroxymyristoyl		2.17	3.73			CH2 1.20-1.32 Terminal CH3 0.82
Lys	7.95	4.11	1.66, 1.52	1.30	1.49	εH 2.72

	CO	αC	βC	γC	δC	
N-3-hydroxymyristoyl	171.33	43.98		36.87		Terminal CH3 14.45
Lys	174.20	52.05	31.13	22.66	26.91	εC 39.11

Table S2. Chemical shifts of synthetic **1** and **2** identified by 1D- and 2D- NMR (gCOSY, gHSQC, gHMBC)

A

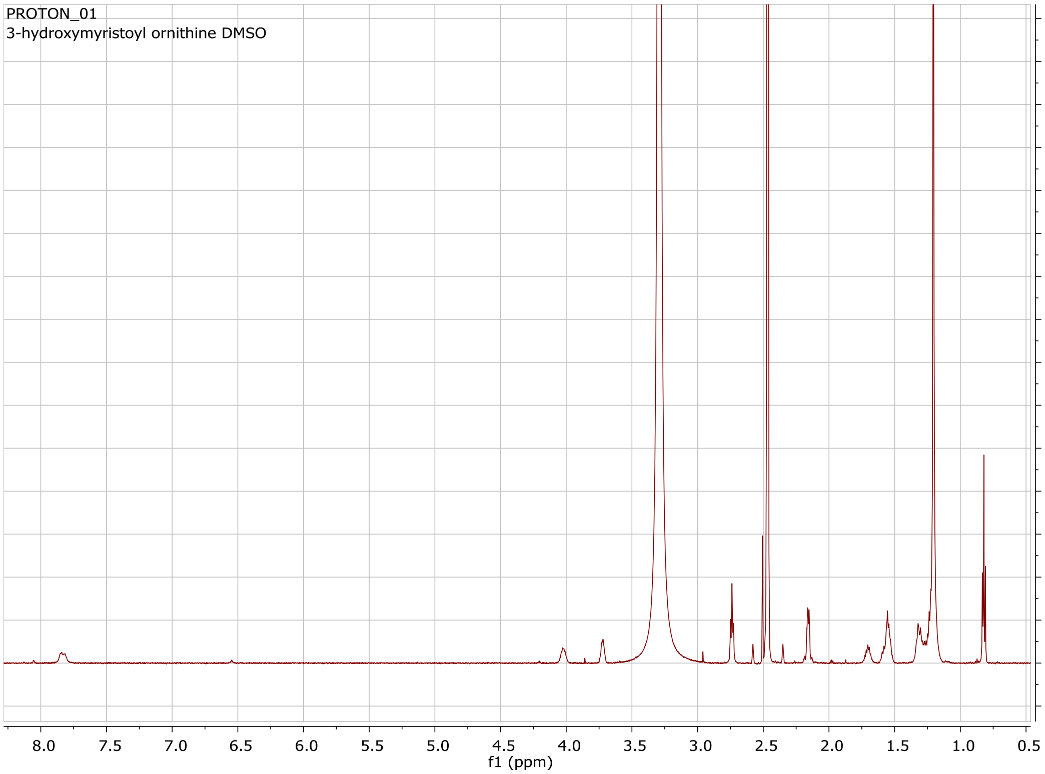
PROTON_01
3-hydroxymyristoyl ornithine DMSO
75
70
65
60
55
50
45
40
35
30
25
20
15
10
5
0
-5
8.0
7.5
7.0
6.5
6.0
5.5
5.0
4.5
4.0
3.5
3.0
2.5
2.0
1.5
1.0
0.5
f1 (ppm)

B

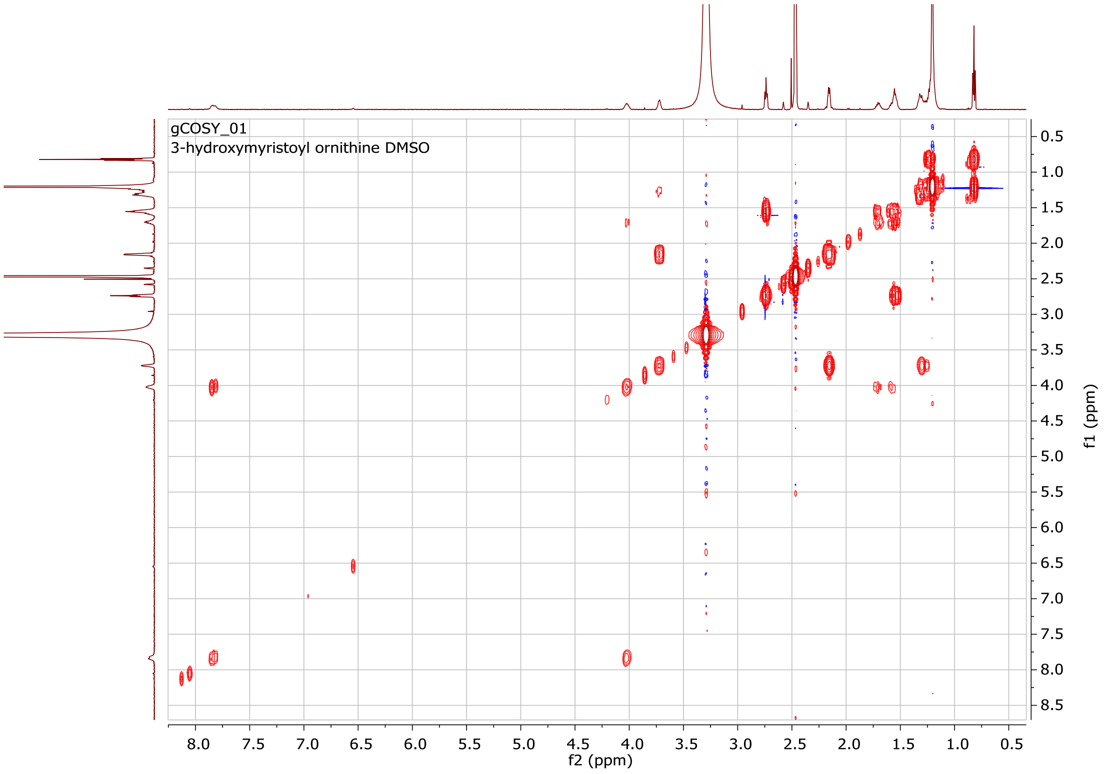
gCOSY_01
3-hydroxymyristoyl ornithine DMSO
f2 (ppm)
f1 (ppm)

C

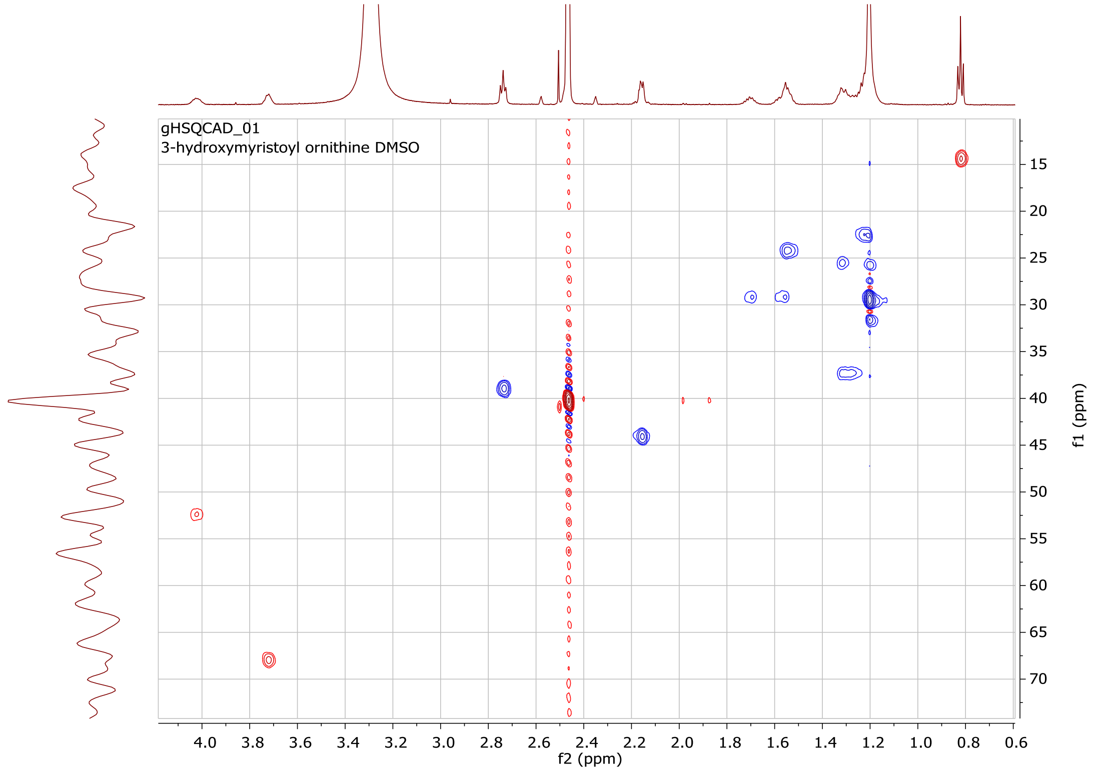
gHSQCAD_01
3-hydroxymyristoyl ornithine DMSO
f2 (ppm)
f1 (ppm)

D

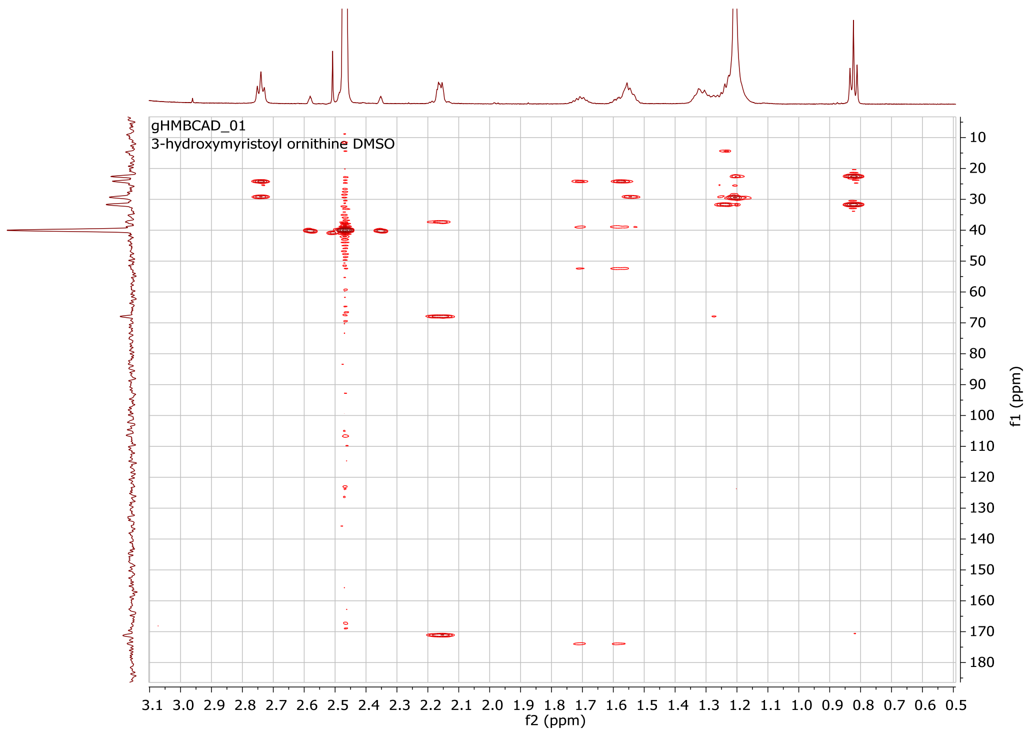

Figure S6. NMR spectra of synthetic compound **1** (DMSO, 600 MHz)

A

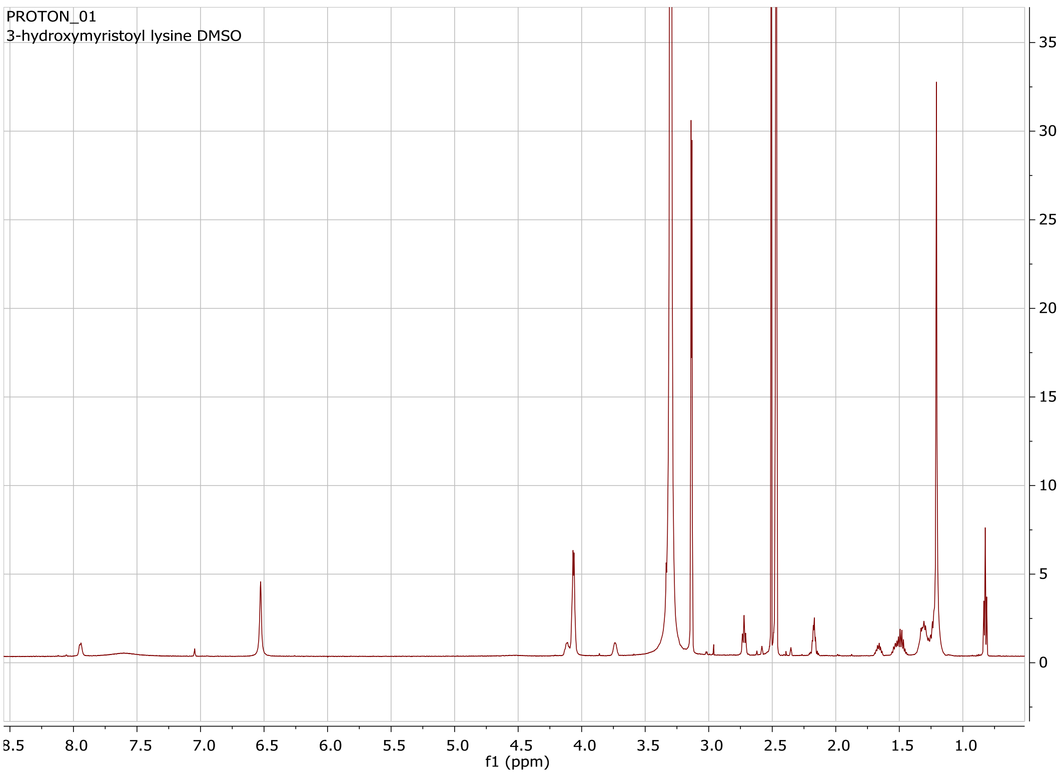

B

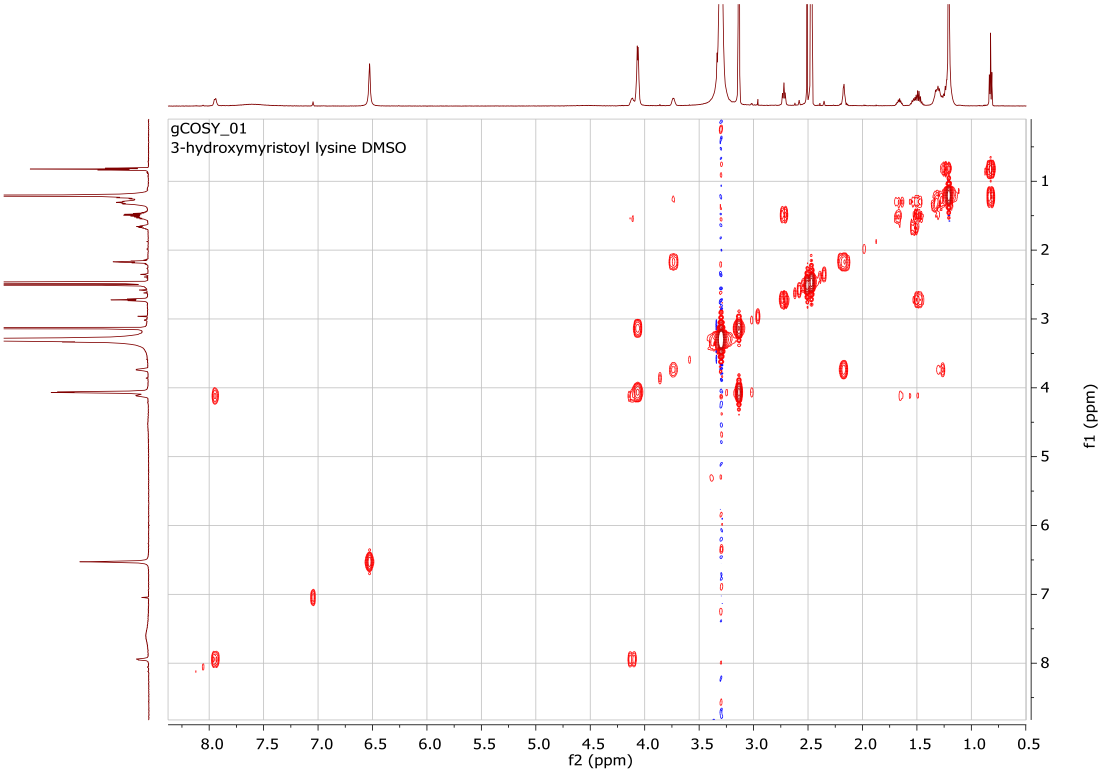

gCOSY_01
3-hydroxymyristoyl lysine DMSO
f1 (ppm)
f2 (ppm)

C

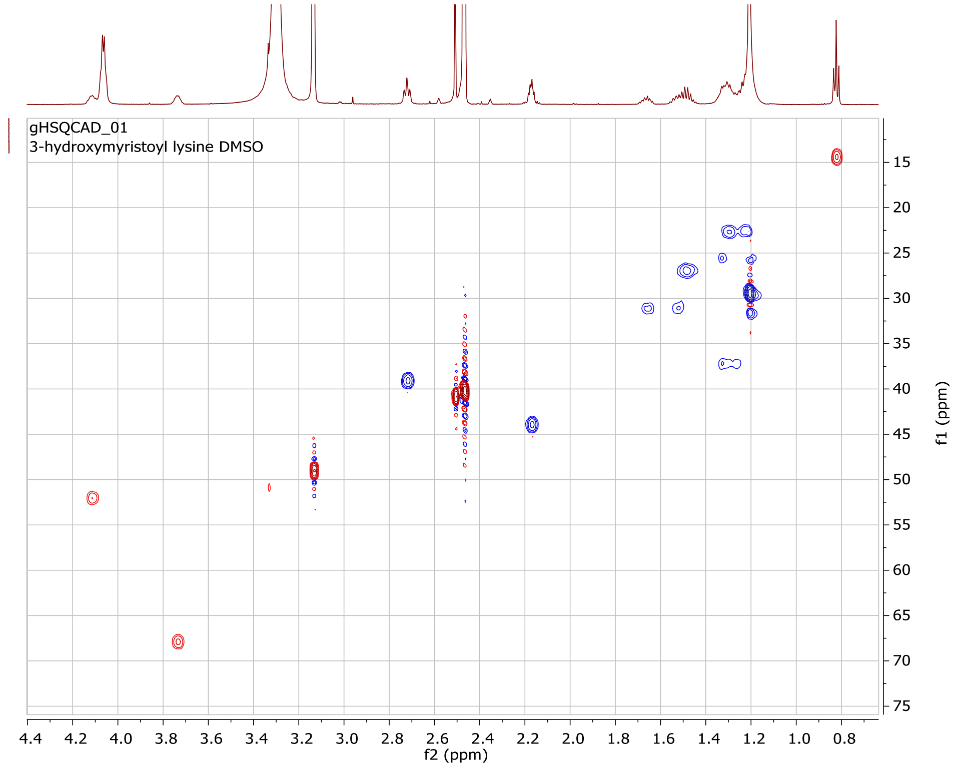

gHSQCAD_01
3-hydroxymyristoyl lysine DMSO
f1 (ppm)
f2 (ppm)

D

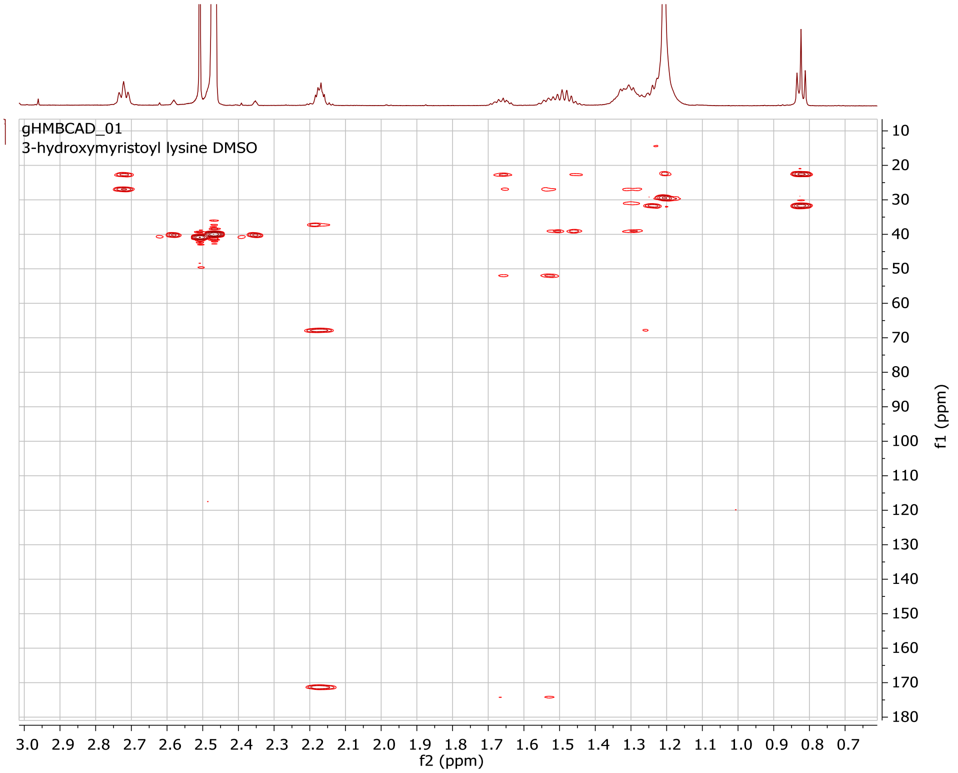

Figure S7. NMR spectra of synthetic compound **2** (DMSO, 600 MHz)

A

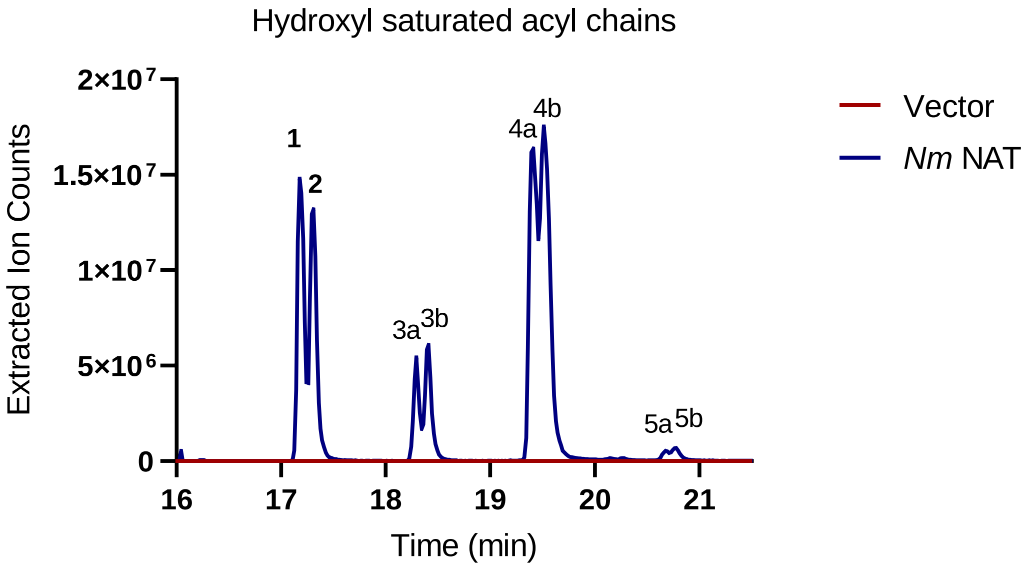

B

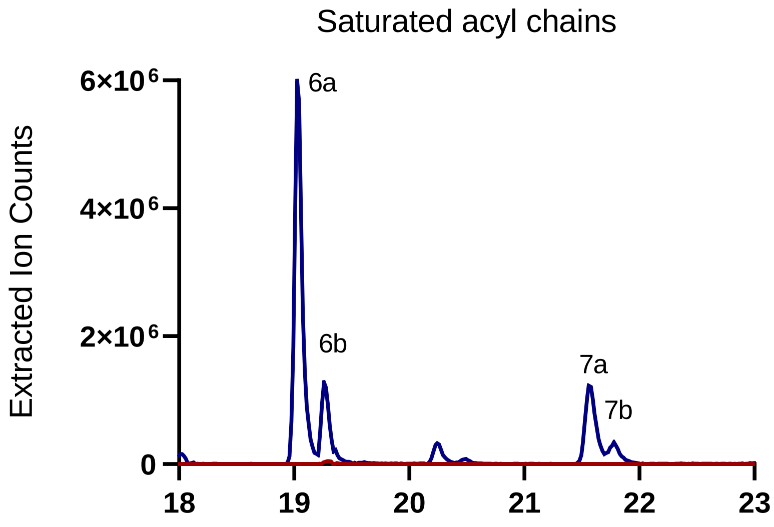
Saturated acyl chains
Extracted Ion Counts
6×10⁶
4×10⁶
2×10⁶
0
6a
6b
7a
7b
18
19
20
21
22
23

C

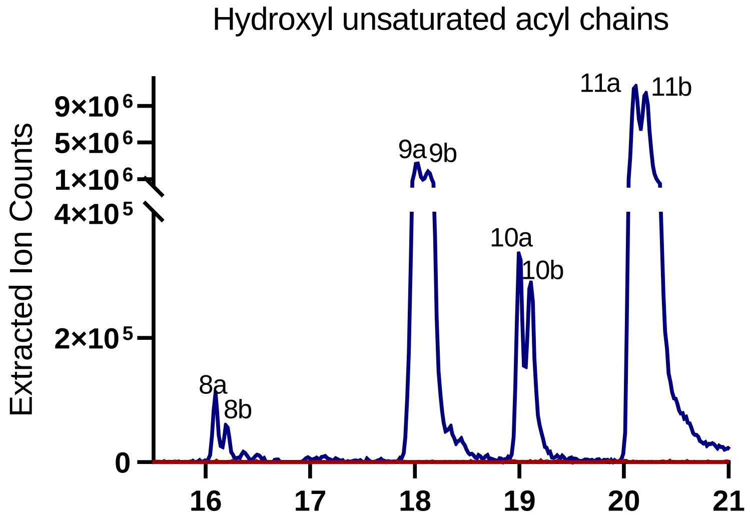
Hydroxyl unsaturated acyl chains
Extracted Ion Counts
9×10⁶
5×10⁶
1×10⁶
4×10⁵
2×10⁵
0
8a
8b
9a
9b
10a
10b
11a
11b
16
17
18
19
20
21

D

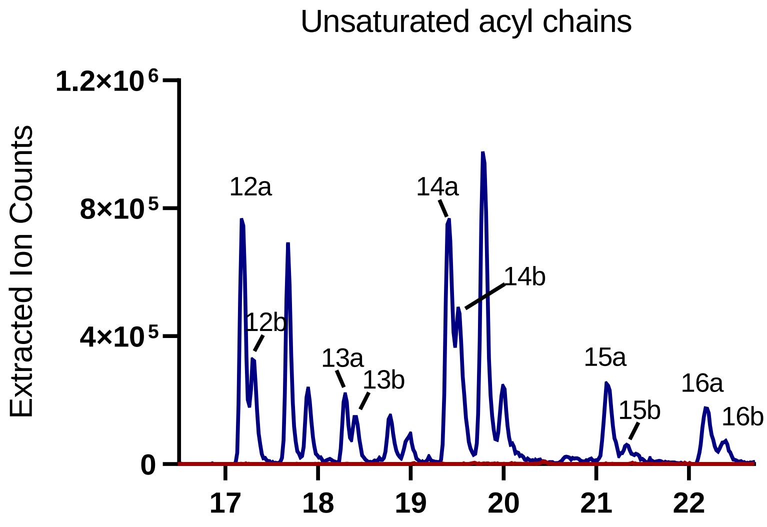

Figure S8. Extracted ion chromatograms of all identified *N*-acyl ornithines and lysines from *Nm*NAT expression in *E. coli*

Head Group	Acyl Chain	Compound	Retention Time (min)	Theoretical *m/z*	Observed *m/z*	Ppm error
Orn						
	OH-C14:0	1	17.177	359.2904	359.2934	8.349792
	OH-C15:0	3a	18.294	373.3061	373.30857	6.616554
	OH-C16:0	4a	19.411	387.3217	387.3246	7.487316
	OH-C17:0	5a	20.678	401.3374	401.33806	1.644502
Lys						
	OH-C14:0	2	17.31	373.3061	373.3086	6.696917
	OH-C15:0	3b	18.41	387.3217	387.3234	4.389116
	OH-C16:0	4b	19.511	401.3374	401.33929	4.709255
	OH-C17:0	5b	20.778	415.353	415.3536	1.444554
Orn						
	C14	6a	19.027	343.2955	343.29833	8.243627
	C16	7a	21.561	371.3268	371.3279	2.96235
Lys						
	C14	6b	19.261	357.3112	357.3132	5.597362
	C16	7b	21.778	385.3425	*385.34231	-0.519019

Orn						
	OH-C14:1	8a	16.093	357.2748	357.2746	-0.559793
	OH-C16:1	9a	18.027	385.3061	385.3071	2.595339
	OH-C17:1	10a	19.011	399.3217	399.3218	0.250425
	OH-C18:1	11a	20.111	413.3374	413.3387	3.14513
Lys						
	OH-C14:1	8b	16.193	371.2904	*371.2915	2.96241
	OH-C16:1	9b	18.127	399.3217	399.3224	1.752973
	OH-C17:1	10b	19.111	413.3374	*413.3376	0.483866
	OH-C18:1	11b	20.211	427.353	427.3548	4.211975
Orn						
	C14:1	12a	17.177	341.2799	341.28111	3.545477
	C15:1	13a	18.294	355.2955	355.2959	1.125823
	C16:1	14a	19.411	369.3112	369.3113	0.270774
	C17:1	15a	21.111	383.3268	383.3278	2.60874
	C18:1	16a	22.195	397.3425	397.3426	0.251672
Lys						
	C14:1	12b	17.293	355.2955	355.2962	1.970191
	C15:1	13b	18.41	369.3112	*369.31151	0.8394
	C16:1	14b	19.527	383.3268	383.3275	1.826118
	C17:1	15b	21.311	397.3425	*397.3427	0.503344
	C18:1	16b	22.395	411.3581	*411.3589	1.944778

Table S3. HRMS of all identified *N*-acyl ornithines and lysines from expression of *Nm*NAT in *E. coli*. Ions marked with * are detected but have inconclusive tandem MS spectra

OH-C14:0 Ornithine

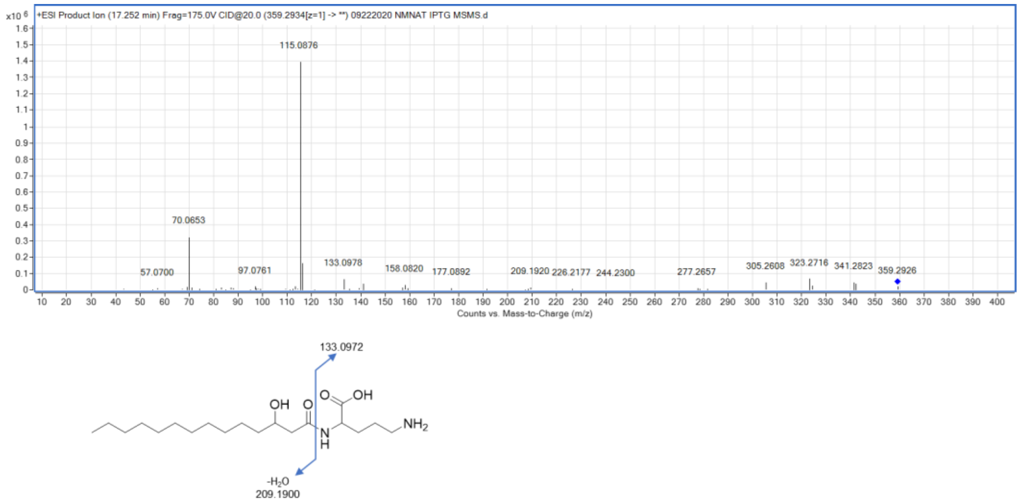

OH-C15:0 Ornithine

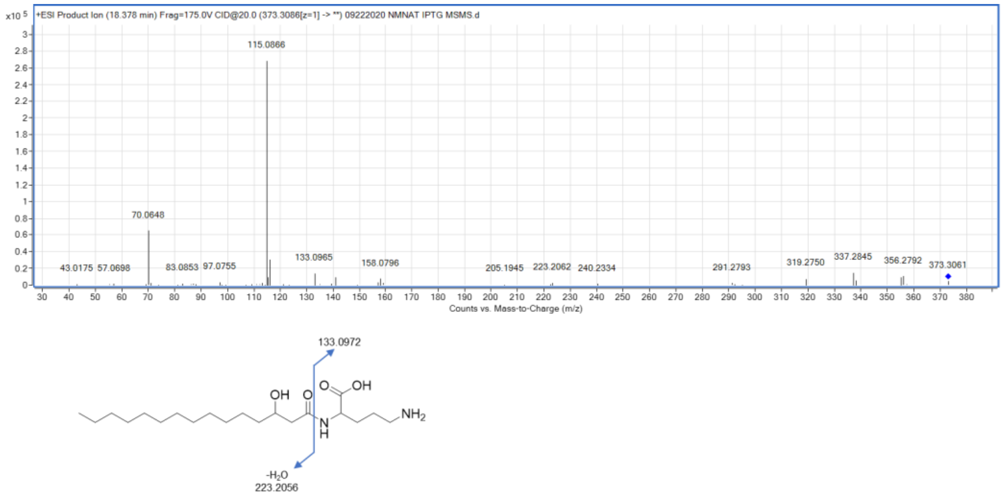

OH-C16:0 Ornithine

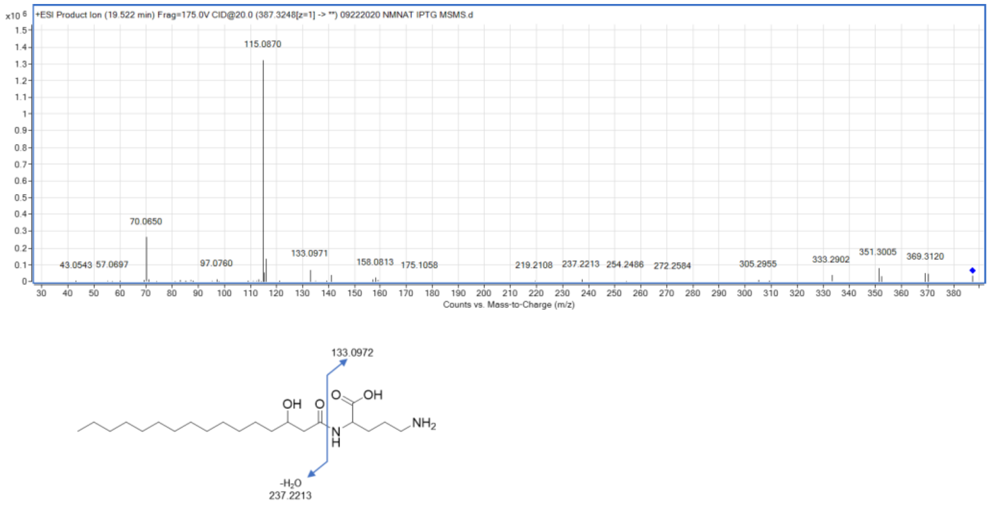

OH-C17:0 Ornithine

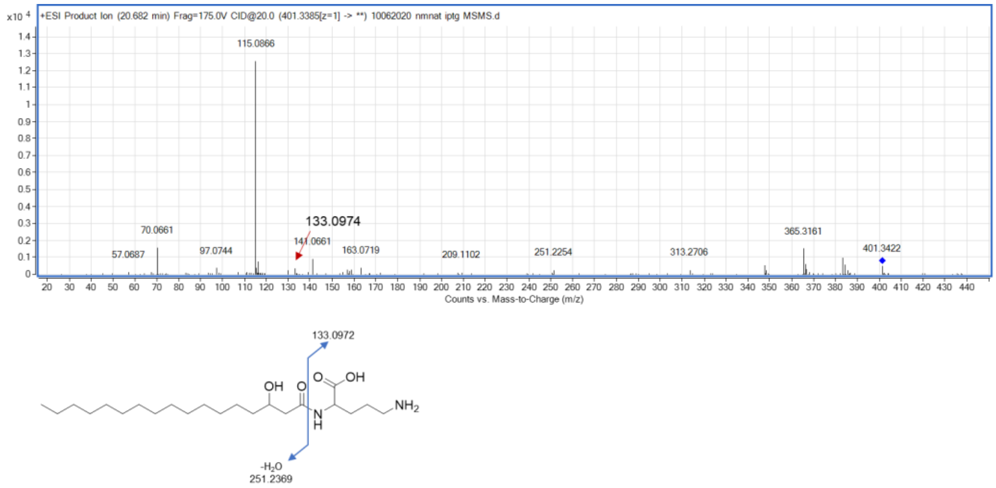

OH-C14:0 Lysine

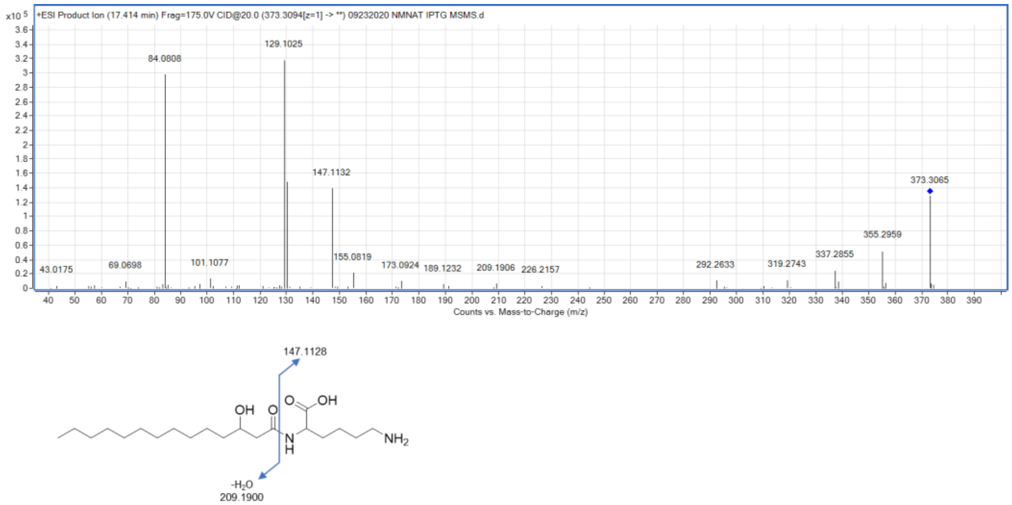

OH-C15:0 Lysine

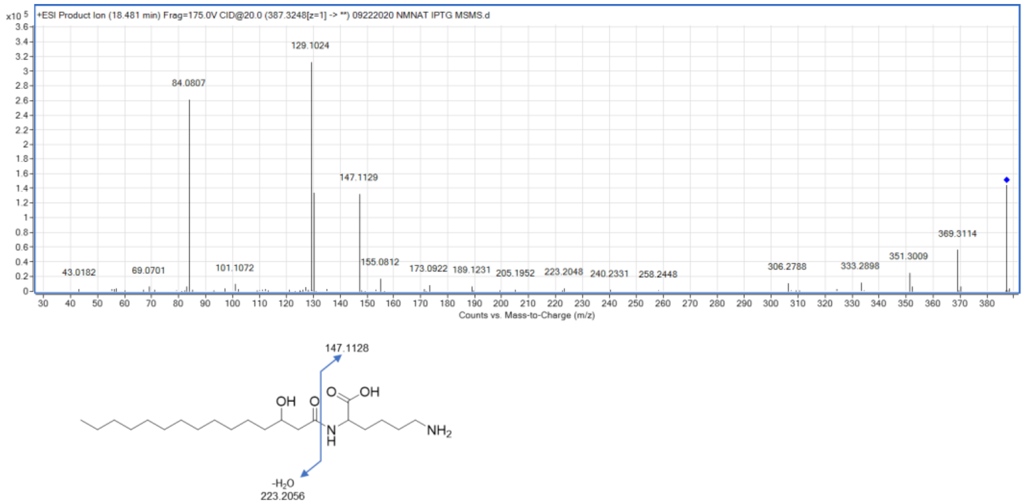

OH-C16:0 Lysine

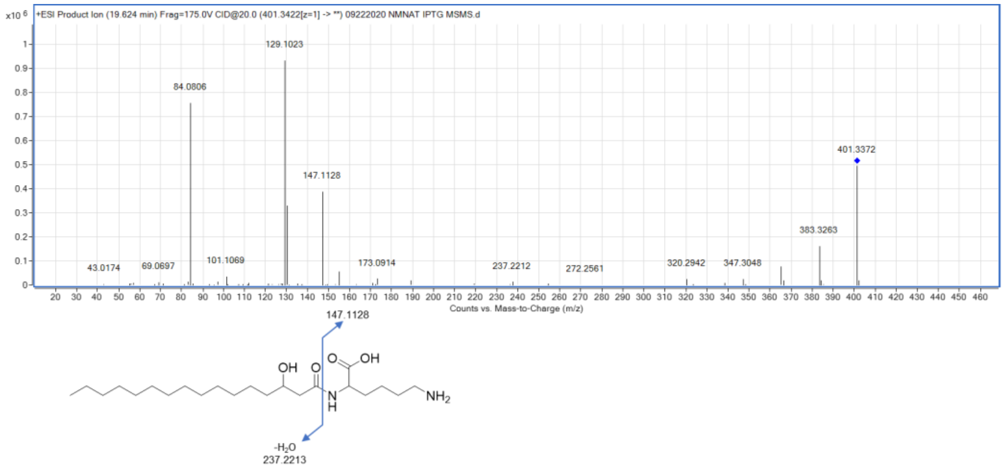

OH-C17:0 Lysine

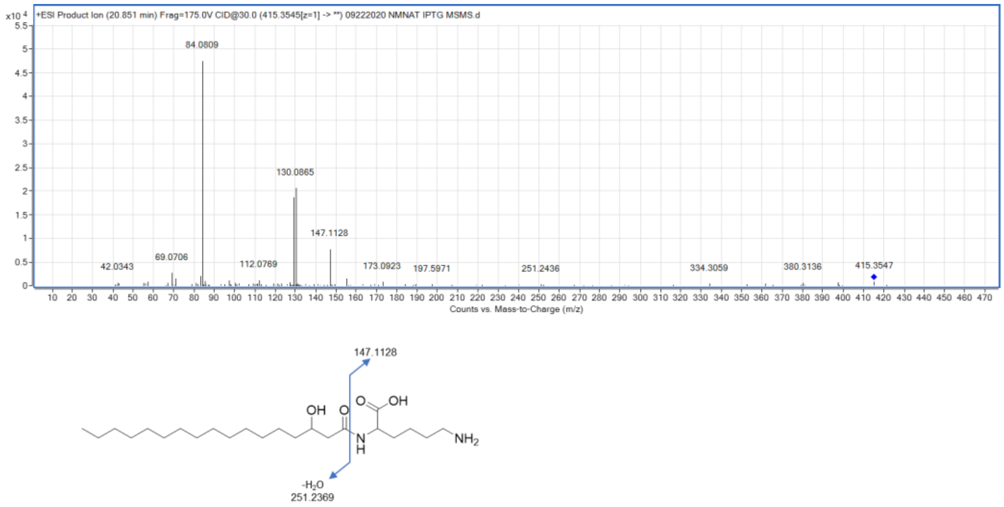

C14:0 Ornithine

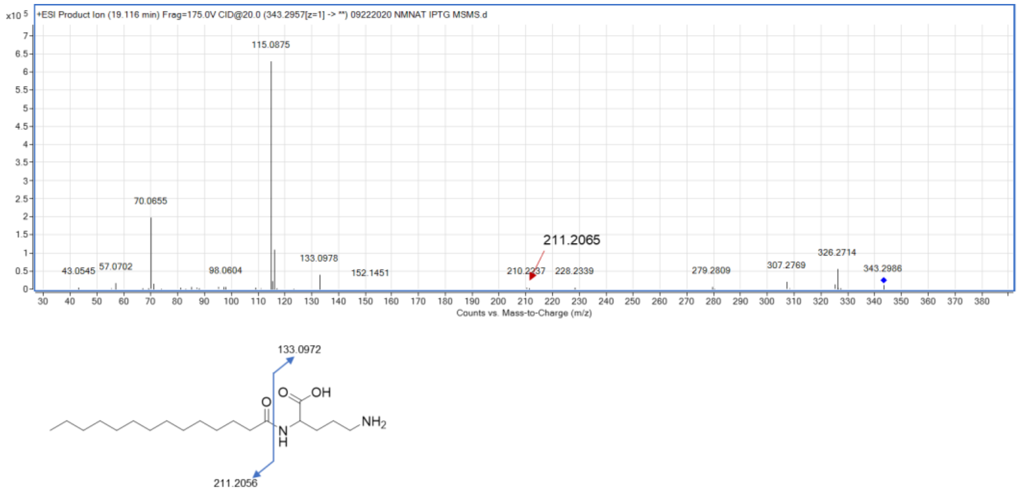

C16:0 Ornithine

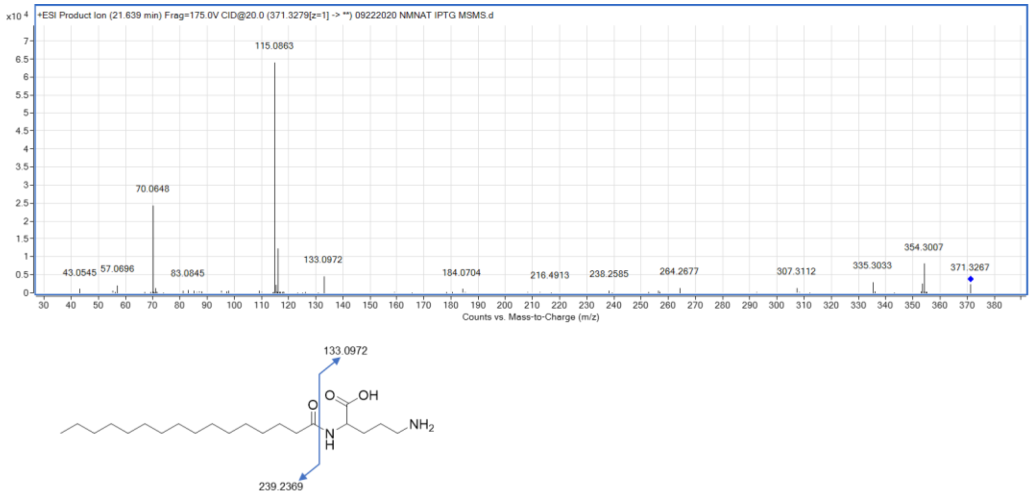

C14:0 Lysine

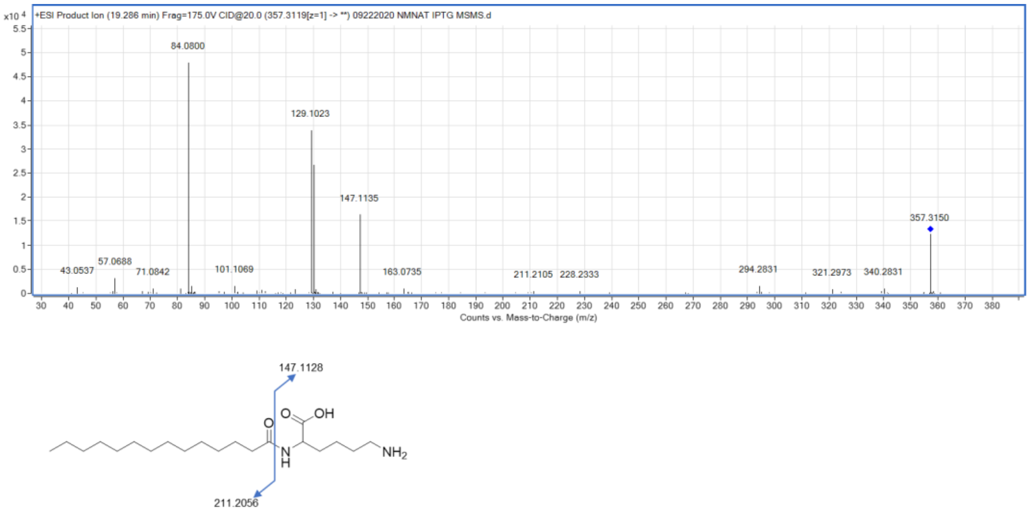

OH-C14:1 Ornithine

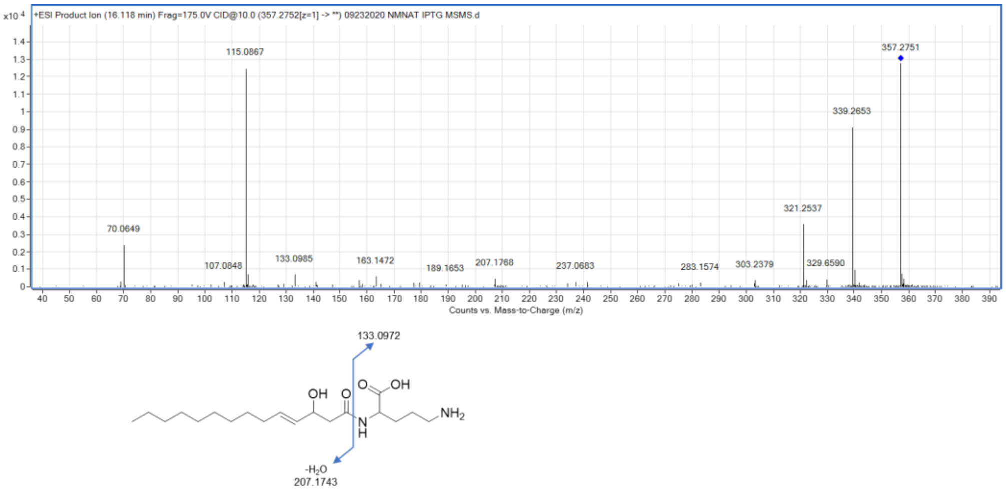

OH-C16:1 Ornithine

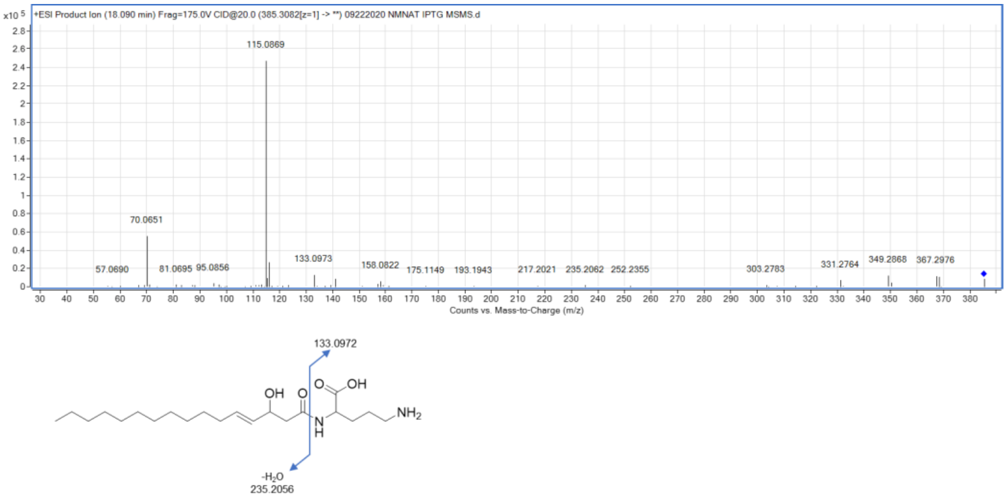

OH-C17:1 Ornithine

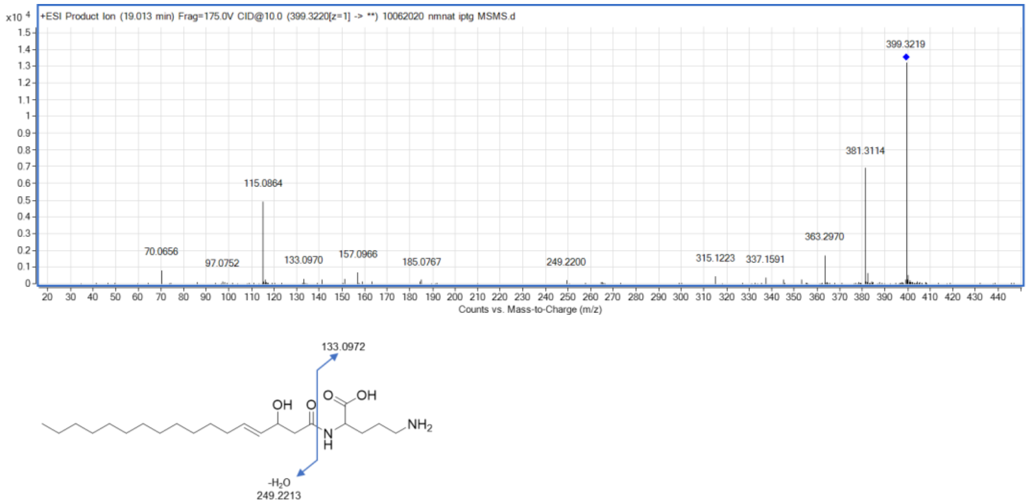

OH-C18:1 Ornithine

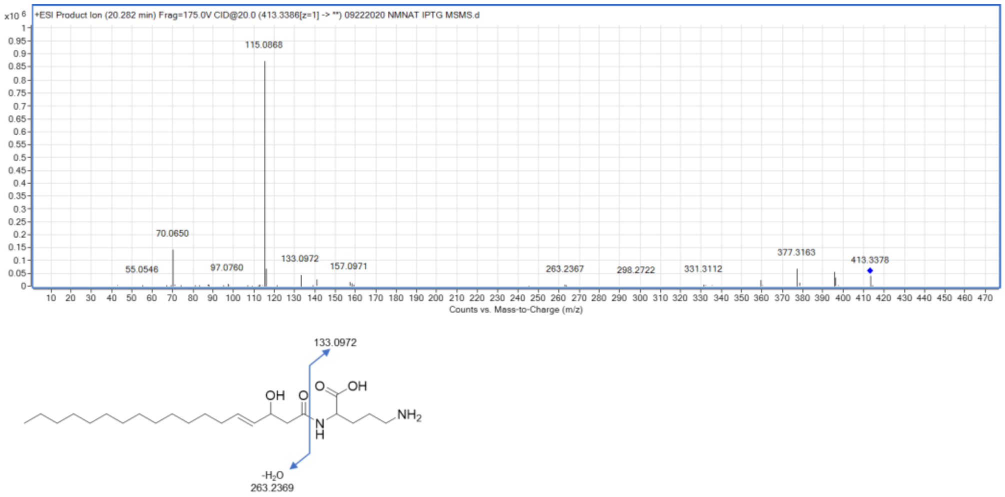

OH-C16:1 Lysine

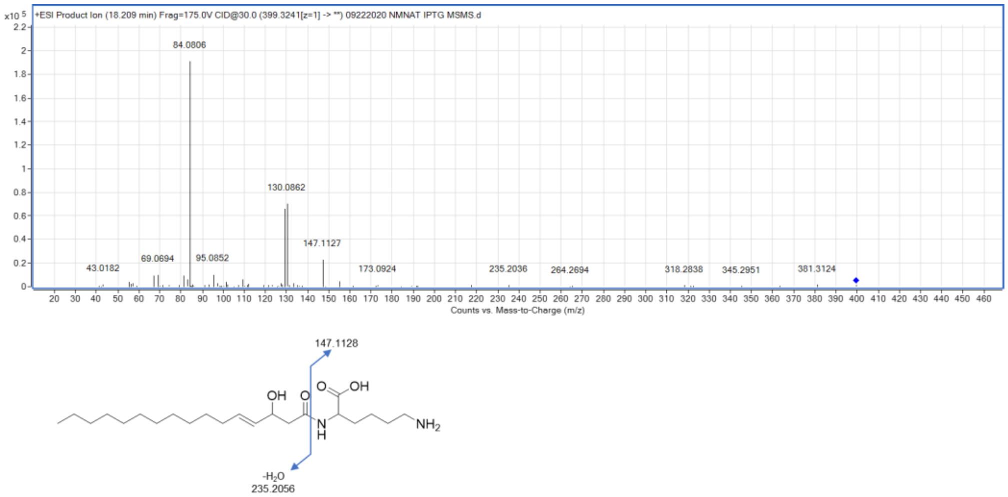

OH-C18:1 Lysine

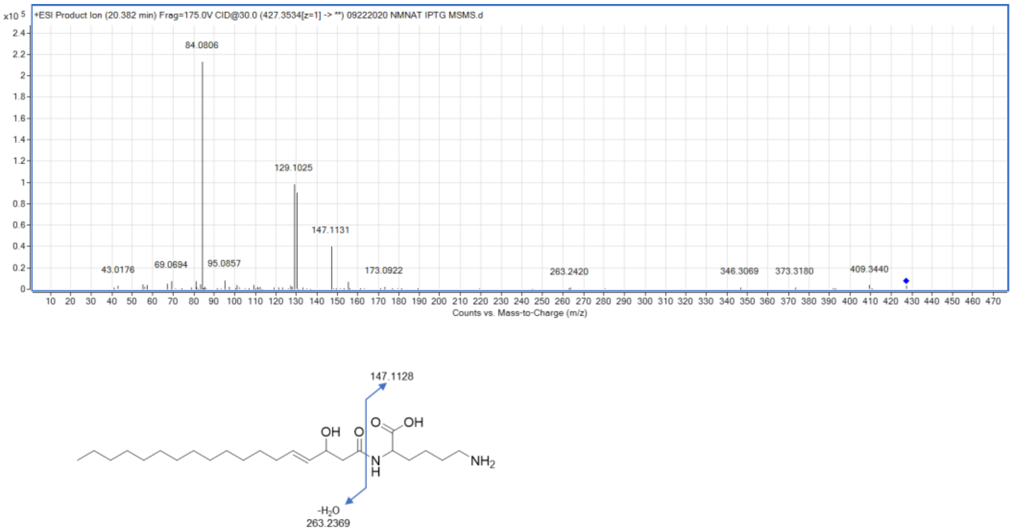

C14:1 Ornithine

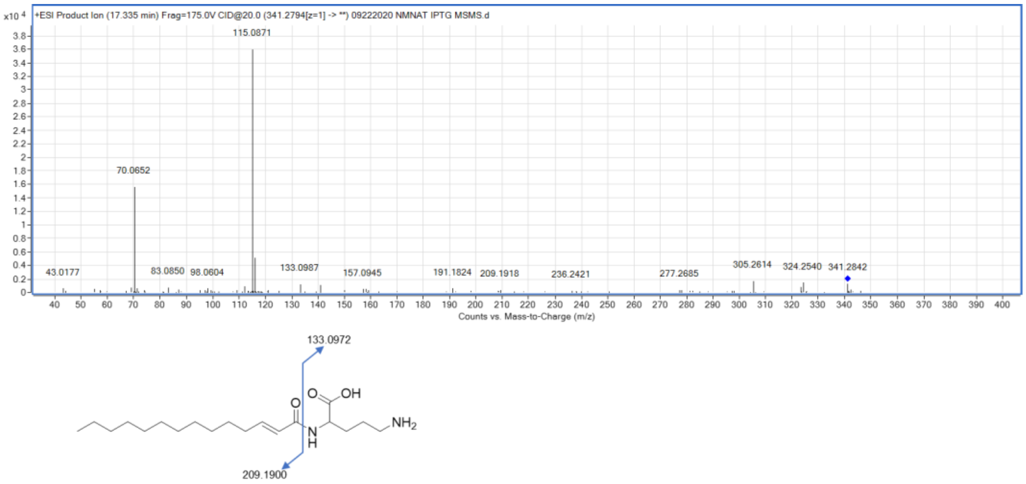

C15:1 Ornithine

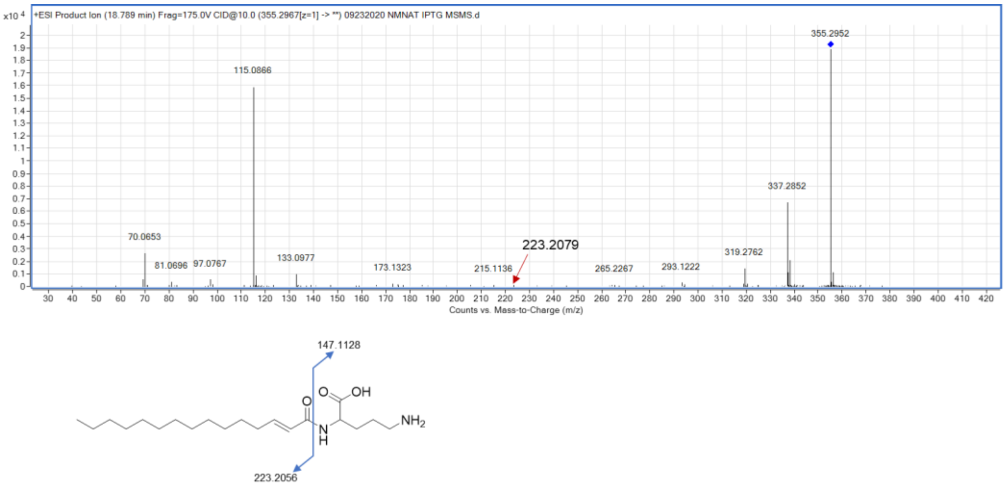

C16:1 Ornithine

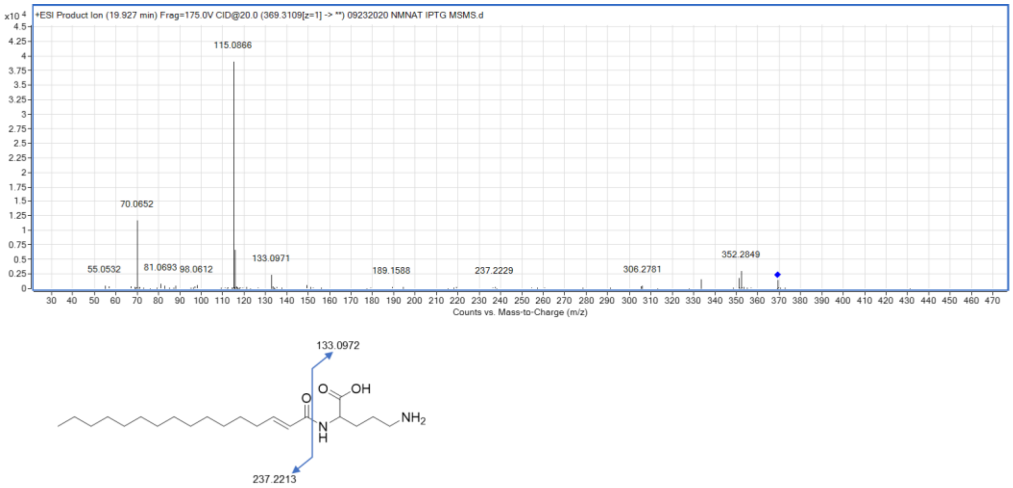

C17:1 Ornithine

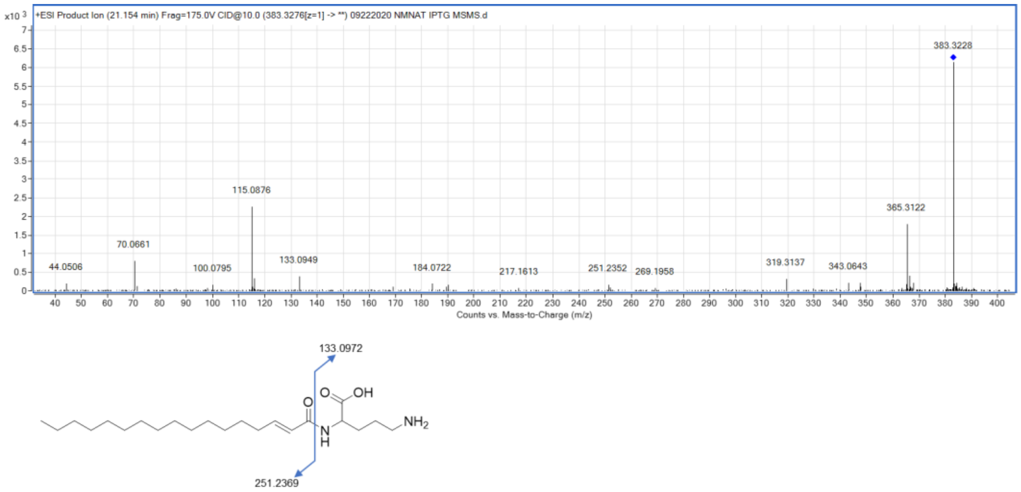

C18:1 Ornithine

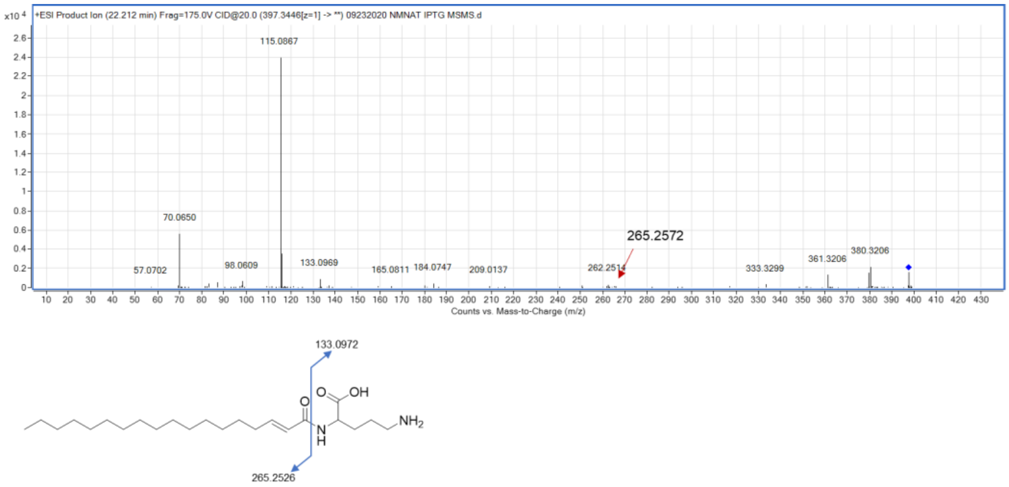

C14:1 Lysine

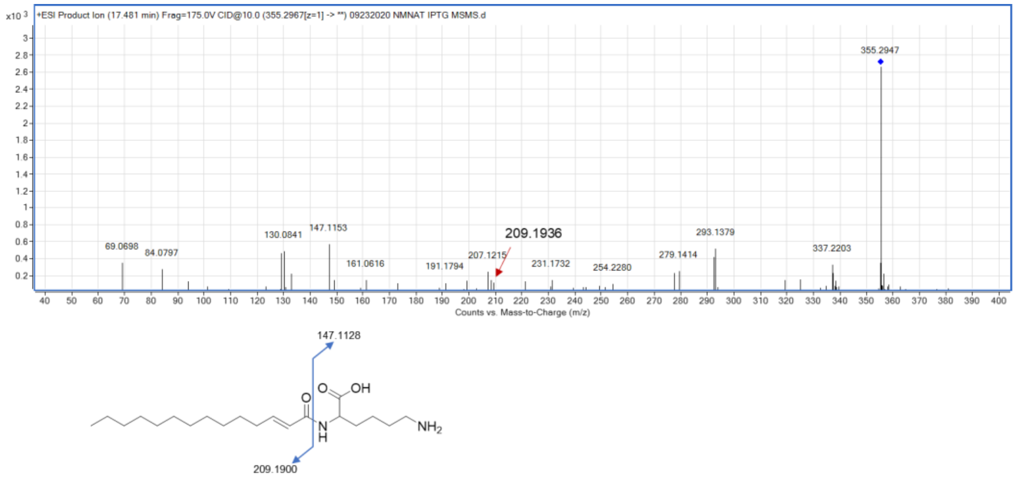

C16:1 Lysine

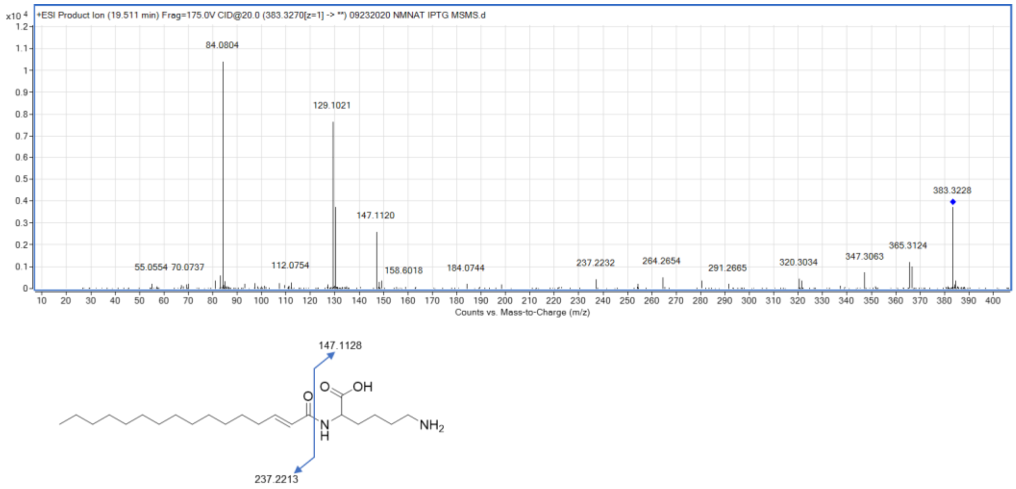

Figure S9. Tandem MS of 24 of the 30 *N*-acyl amides detected from *Nm*NAT expression. Corresponding structures show deduced structures, where regiochemistry of hydroxyl groups and unsaturation, as well as stereochemistry of points of unsaturation are assumed. The straight acyl chain is also a simplification, as they may occur as branched chain fatty acids.

Structural elucidation of 3 and 4

Compound	Molecular Formula	Theoretical *m/z*	Observed *m/z*	Ppm error
3	$C_{16}H_{31}NO_3$	286.2377	286.2381	1.39744
4	$C_{19}H_{29}NO_3$	320.222	320.2229	2.81055

Table S4. Theoretical and observed HRMS values of compounds **3** and **4**

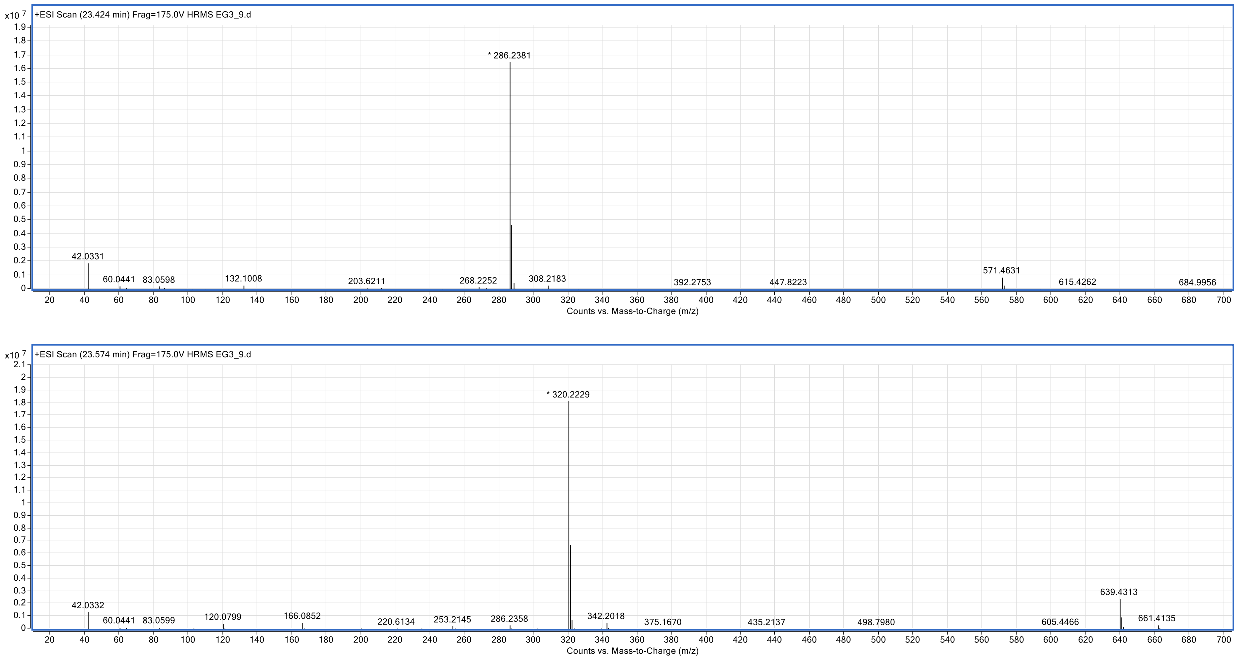

Figure S10. HRMS of compounds **3** and **4**

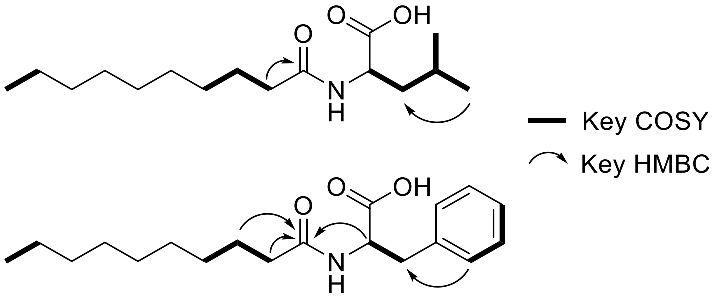

Figure S11. Key COSY and HMBC correlations of natural **3** and **4** identified from a mixture

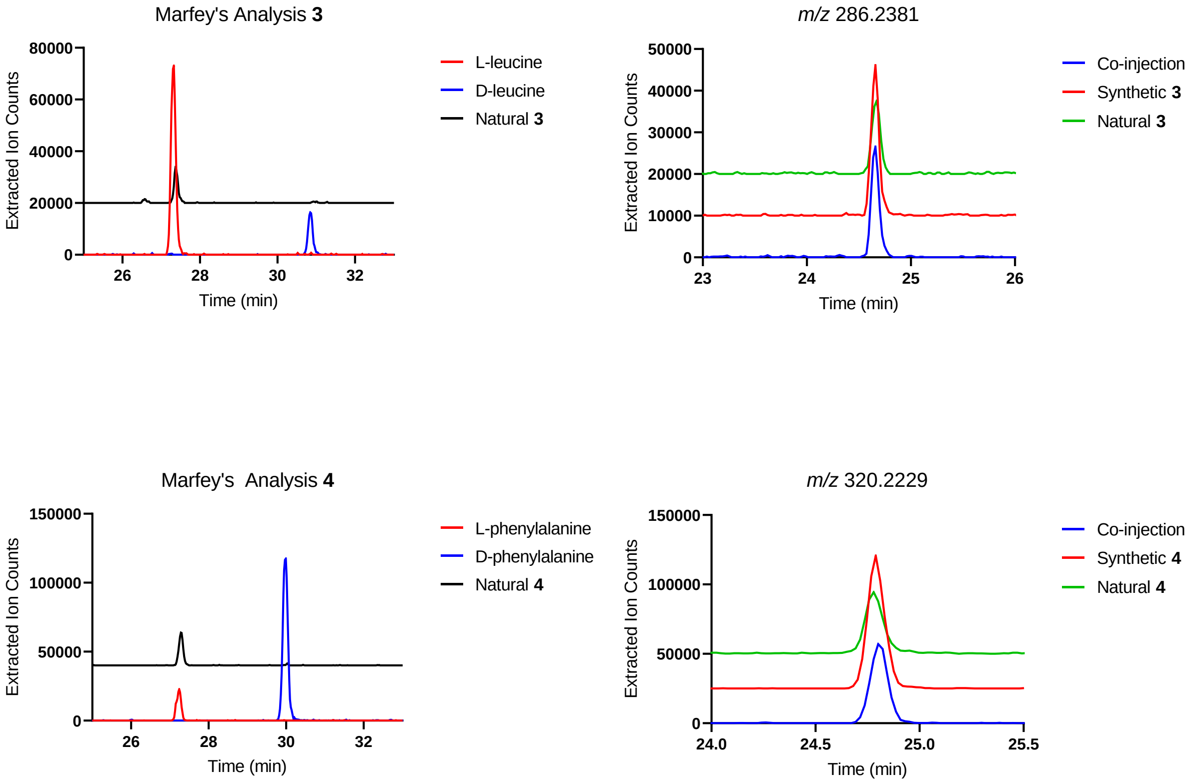

Figure S12. Marfey's analysis for stereochemical elucidation of compounds **3** and **4** (left). Co-injection with synthetic **3** and **4** for validation of structure (right).

***N*-decanoyl-L-leucine (MH+ 286)** (CDCl3, 400 MHz)

	NH	αH	βH	γH	δH	
N-decanoyl		2.26	1.64			CH2 1.25-1.35<br>Terminal CH3 0.89
Leu	8.05	4.62	1.74<br>1.60	1.73	0.97	

	CO	αC	βC	γC	δC	
N-decanoyl	174.17	36.50	25.61			CH2 21.86, 29.19-31.86<br>Terminal CH3 14.07
Leu	176.39	50.93	41.16	24.91	22.65<br>22.83	

Table S5. Chemical shifts of synthetic **3** as identified by 1D- (^{1}H and ^{13}C) and 2D- (gCOSY) NMR

***N*-decanoyl-L-phenylalanine (MH+ 320)** (DMSO, 400 MHz)

	NH	αH	βH		
N-decanoyl		2.03	1.39	CH2 1.08-1.32<br>Terminal CH3 0.87	
Phe	8.07	4.43	2.84<br>3.06	Aromatic<br>7.17-7.29	12.65<br>COOH

	**CO**	**αC**	**βC**	
N-decanoyl	172.57	35.53	25.63	CH2 22.55-31.69<br>Terminal CH3 14.39
Phe	173.69	53.77	37.24	Aromatic<br>126.77-138.26

Table S6. Chemical shifts of synthetic **3** as identified by 1D- (^{1}H and ^{13}C) and 2D- (gCOSY) NMR

A

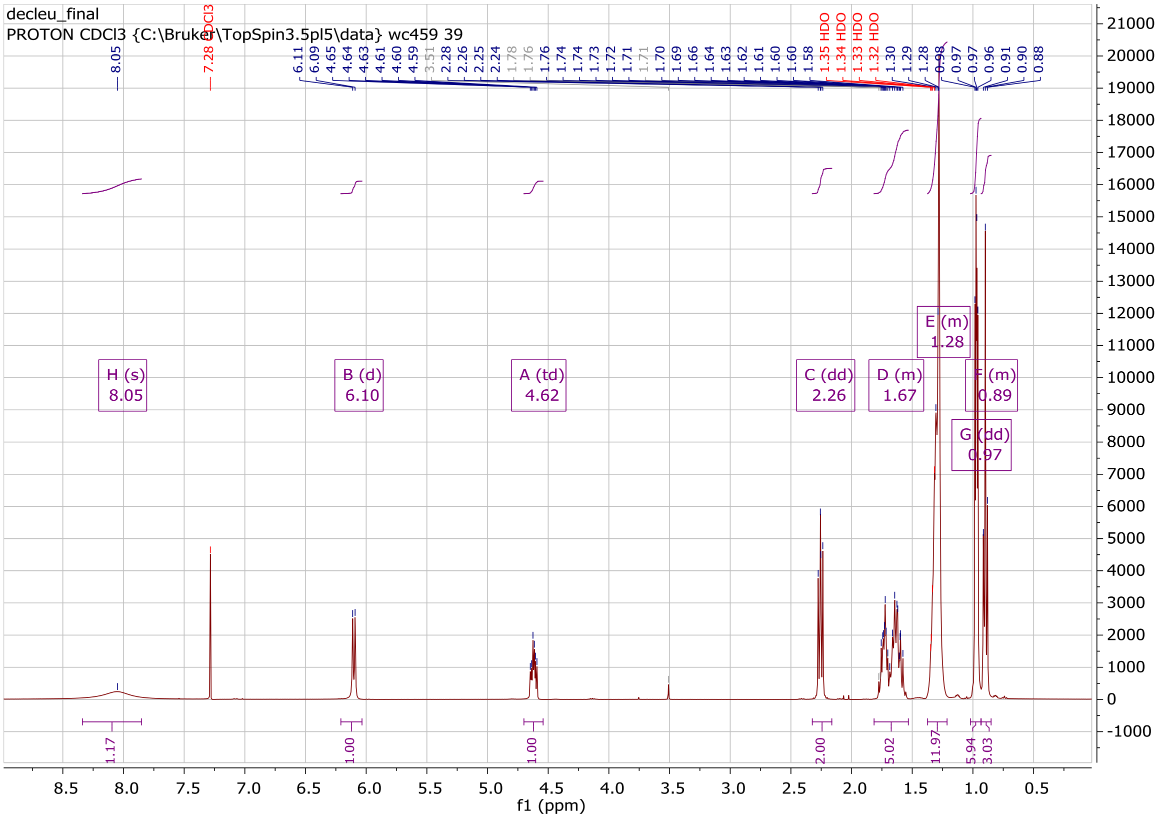

B

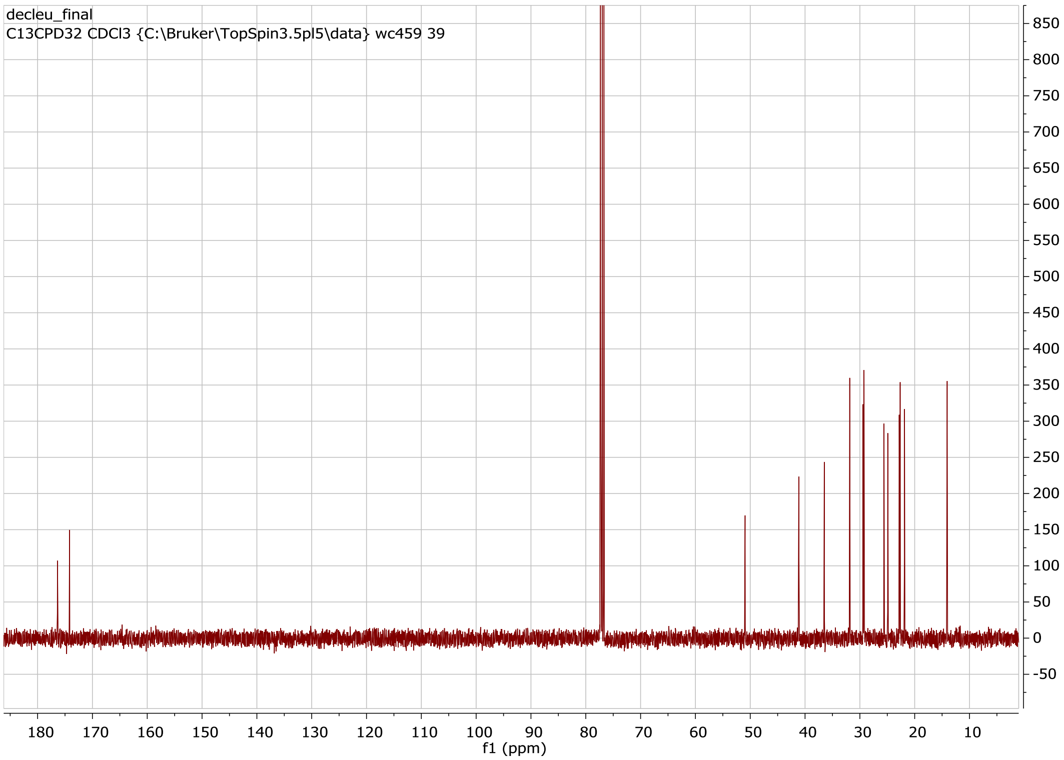

C

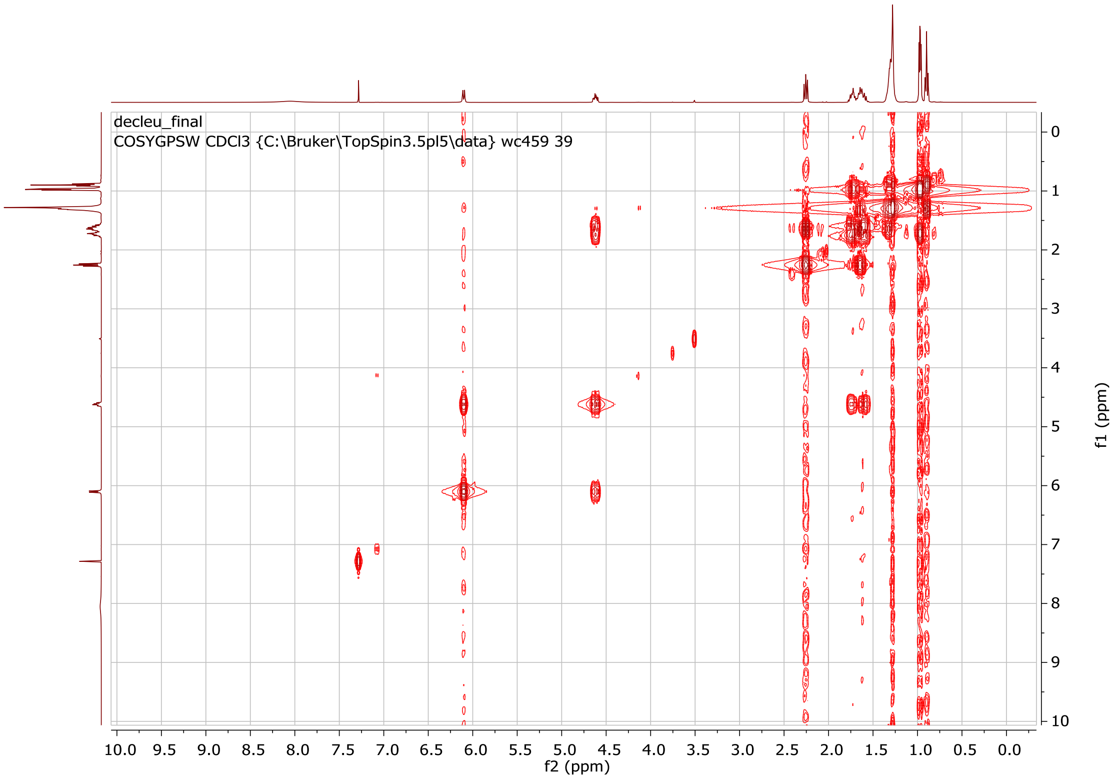

Figure S13. NMR spectra of synthetic compound **3** (CDCl3, 400 MHz)

A

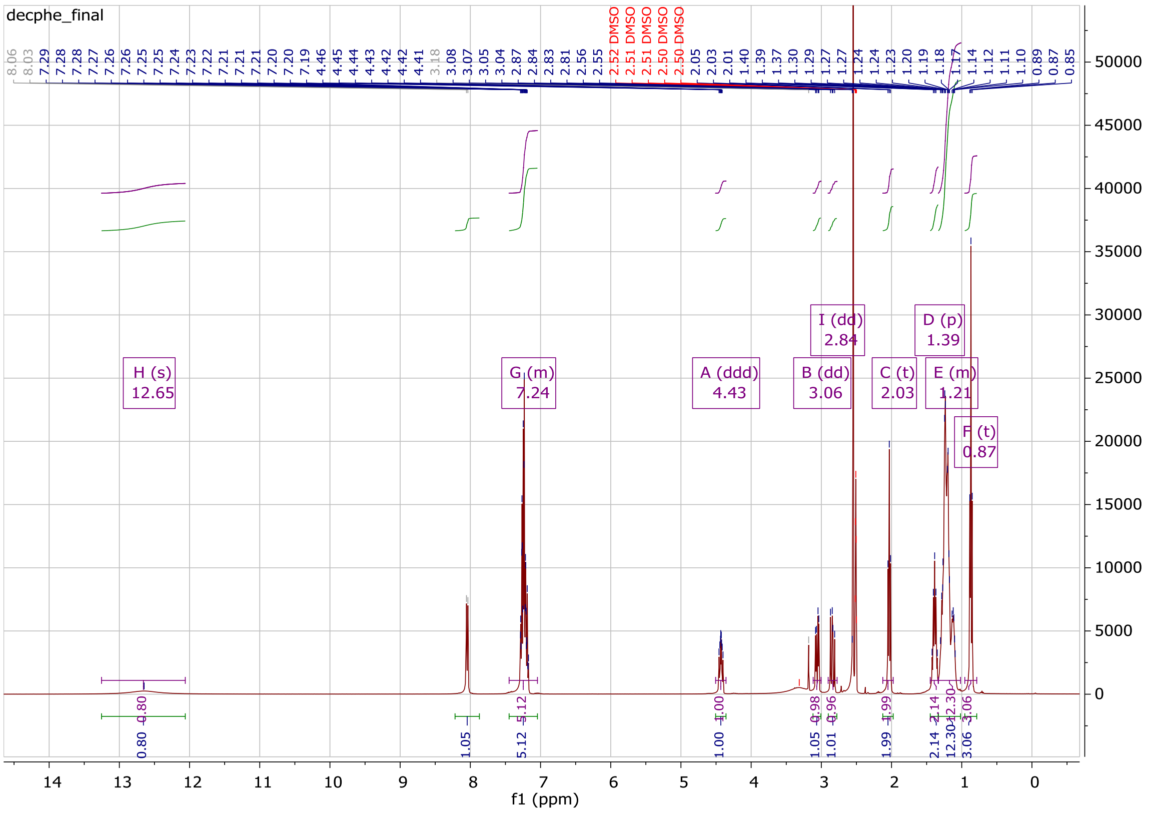
decphe_final
8.06
8.03
7.29
7.28
7.28
7.27
7.26
7.26
7.25
7.25
7.24
7.23
7.22
7.21
7.21
7.21
7.20
7.20
7.19
4.46
4.45
4.44
4.43
4.42
4.42
4.41
3.18
3.08
3.07
3.05
3.04
2.87
2.84
2.83
2.81
2.56
2.55
2.52 DMSO
2.51 DMSO
2.51 DMSO
2.50 DMSO
2.50 DMSO
2.05
2.03
2.01
1.40
1.39
1.37
1.30
1.29
1.27
1.27
1.24
1.24
1.23
1.20
1.19
1.18
1.17
1.14
1.12
1.11
1.10
0.89
0.87
0.85
H (s)
12.65
G (m)
7.24
A (ddd)
4.43
B (dd)
3.06
I (dd)
2.84
C (t)
2.03
D (p)
1.39
E (m)
1.21
F (t)
0.87
0.80
1.05
5.12
1.00
1.05
0.98
1.01
0.96
1.99
2.14
12.30
3.06
50000
45000
40000
35000
30000
25000
20000
15000
10000
5000
0
14
13
12
11
10
9
8
7
6
5
4
3
2
1
0
f1 (ppm)

B

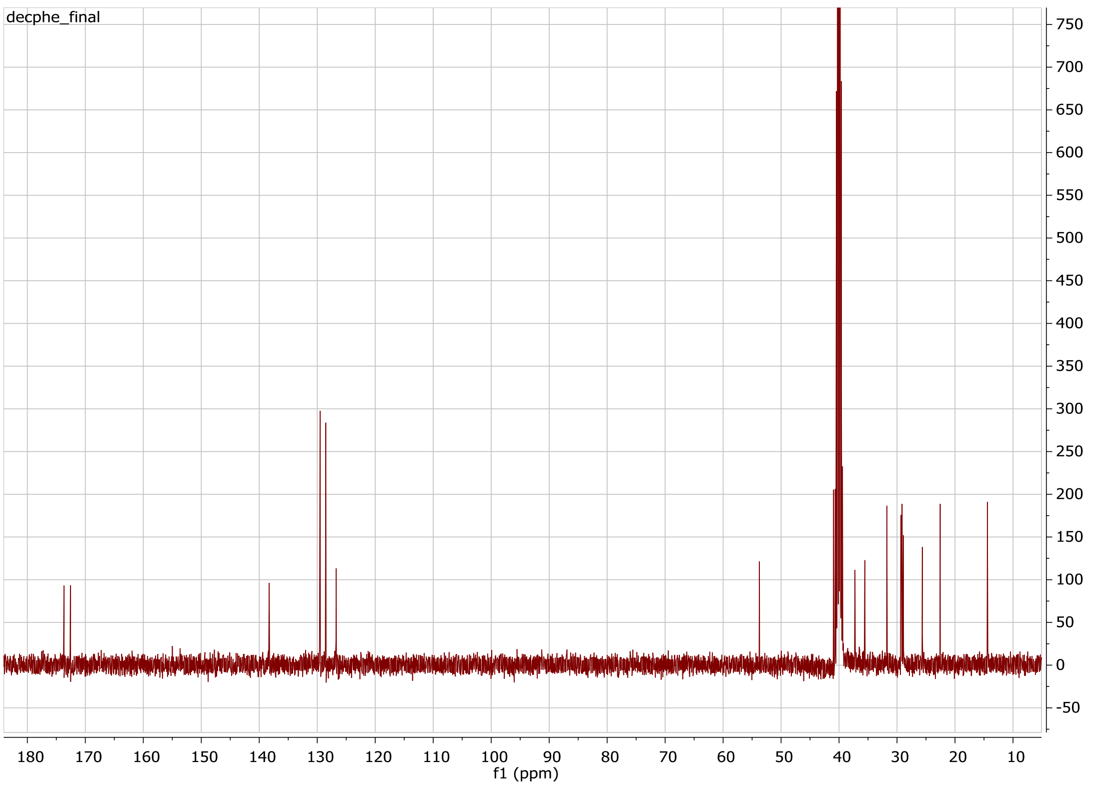
decphe_final
750
700
650
600
550
500
450
400
350
300
250
200
150
100
50
0
-50
180
170
160
150
140
130
120
110
100
90
80
70
60
50
40
30
20
10
f1 (ppm)

C

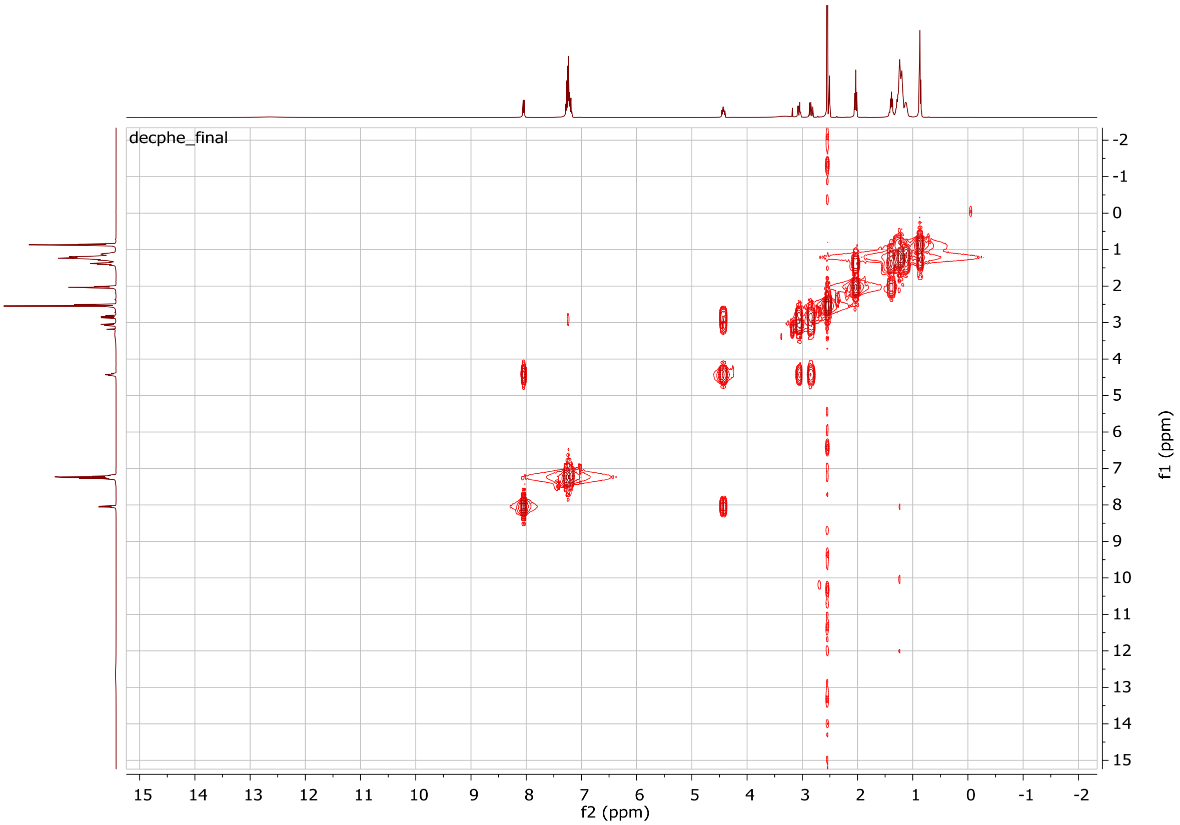

Figure S14. NMR spectra of synthetic compound **4** (DMSO, 600 MHz)

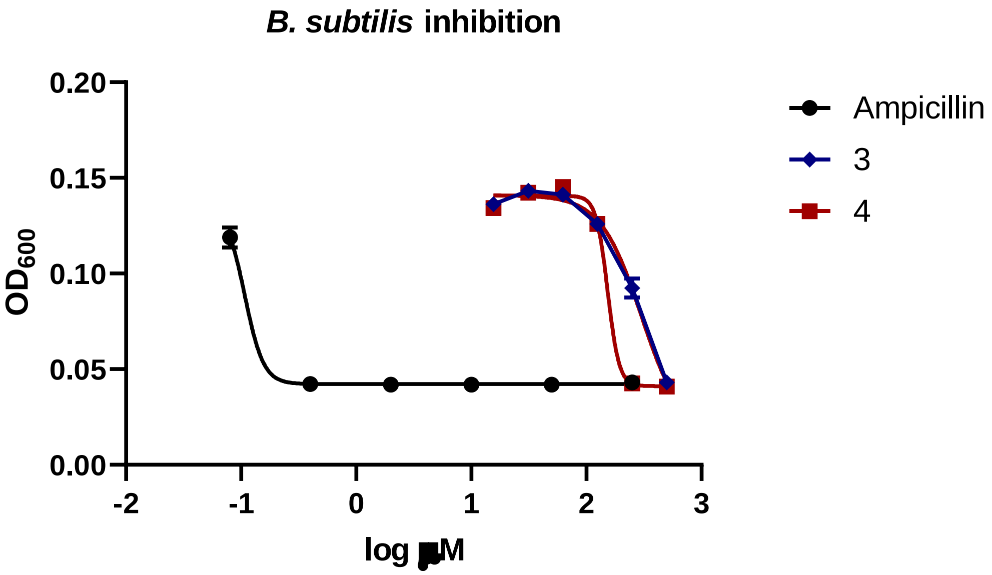

Figure S15. Antibacterial assay of compounds **3** and **4** against *Bacillus subtilis*. IC_{50} was calculated as 301.2 μM and 153 μM for **3** and **4**, respectively, through a variable slope (four parameters) fitting on GraphPad Prism.

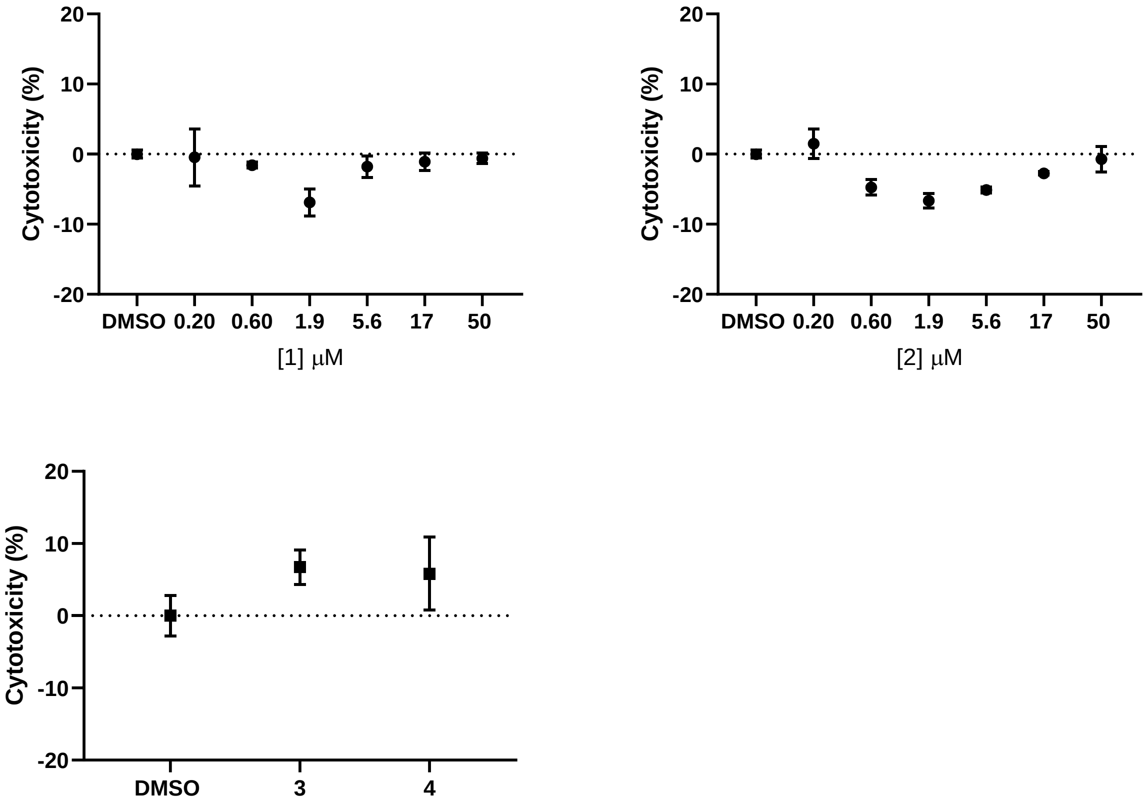

Figure S16. Cytotoxicity (LDH readout) measurements of compounds **1** and **2** at varying concentrations, and of compounds **3** and **4** at fixed concentration of 30 μM.
